# All‐In‐One OsciDrop Digital PCR System for Automated and Highly Multiplexed Molecular Diagnostics

**DOI:** 10.1002/advs.202309557

**Published:** 2024-03-22

**Authors:** Caiming Li, Nan Kang, Shun Ye, Weihang Huang, Xia Wang, Cheng Wang, Yuchen Li, Yan‐Fei Liu, Ying Lan, Liang Ma, Yuhang Zhao, Yong Han, Jun Fu, Danhua Shen, Lianhua Dong, Wenbin Du

**Affiliations:** ^1^ State Key Laboratory of Microbial Resources Institute of Microbiology Chinese Academy of Sciences Beijing 100101 China; ^2^ College of Life Sciences University of the Chinese Academy of Sciences Beijing 101408 China; ^3^ Department of Pathology Peking University People's Hospital Beijing 100044 China; ^4^ Department of Bioengineering University of California Los Angeles Los Angeles CA 90095 USA; ^5^ Center for Corpus Research Department of English Language and Linguistics University of Birmingham Edgbaston Birmingham B152TT UK; ^6^ Center for Advanced Measurement Science National Institute of Metrology Beijing 100013 China; ^7^ Department of Breast Surgery Huangpu Branch Shanghai Ninth People's Hospital Affiliated to Shanghai Jiao Tong University School of Medicine Shanghai 200011 China; ^8^ Biomedical Sciences College & Shandong Medical Biotechnology Centre Shandong First Medical University & Shandong Academy of Medical Sciences Jinan 250000 China; ^9^ Research Center for Analytical Sciences Northeastern University Shenyang 110819 China; ^10^ Maccura Biotechnology Co., Ltd Chengdu 611730 China; ^11^ Savaid Medical School University of the Chinese Academy of Sciences Beijing 101408 China

**Keywords:** biomedical engineering, chip‐free microfluidics, digital PCR, molecular diagnostics, multiplex nucleic acid testing

## Abstract

Digital PCR (dPCR) holds immense potential for precisely detecting nucleic acid markers essential for personalized medicine. However, its broader application is hindered by high consumable costs, complex procedures, and restricted multiplexing capabilities. To address these challenges, an all‐in‐one dPCR system is introduced that eliminates the need for microfabricated chips, offering fully automated operations and enhanced multiplexing capabilities. Using this innovative oscillation‐induced droplet generation technique, OsciDrop, this system supports a comprehensive dPCR workflow, including precise liquid handling, pipette‐based droplet printing, in situ thermocycling, multicolor fluorescence imaging, and machine learning‐driven analysis. The system's reliability is demonstrated by quantifying reference materials and evaluating *HER2* copy number variation in breast cancer. Its multiplexing capability is showcased with a quadruplex dPCR assay that detects key *EGFR* mutations, including 19Del, L858R, and T790M in lung cancer. Moreover, the digital stepwise melting analysis (dSMA) technique is introduced, enabling high‐multiplex profiling of seven major *EGFR* variants spanning 35 subtypes. This innovative dPCR system presents a cost‐effective and versatile alternative, overcoming existing limitations and paving the way for transformative advances in precision diagnostics.

## Graphical abstract

This work unveils an innovative, chip‐free digital PCR system, revolutionizing precision diagnostics by overcoming cost and complexity barriers. The OsciDrop technique, paired with advanced instrumentation and machine learning, enables highly sensitive, efficient nucleic acid quantification. This system streamlines dPCR, introduces digital stepwise melting analysis, and significantly boosts multiplexing and ease of use, paving the way for scalable, cost‐effective diagnostics.

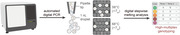

## 1 Introduction

In precision medicine, the role of molecular diagnostics is critical, particularly in its capacity to assess unique nucleic acid biomarkers, encompassing infectious agents, gene mutations, and epigenetic modifications relevant to illnesses and therapeutic interventions.^[^ ^1^ ^]^ Of its various techniques, polymerase chain reaction (PCR) emerges as the cornerstone, essential for genetic research and clinical diagnostics. The advent of digital PCR (dPCR), the third generation of PCR technology, has particularly revolutionized precision diagnostics by partitioning samples into numerous nanoliter compartments, amplifying target templates, and calculating absolute template quantities based on the Poisson distribution.^[^ ^2^, ^3^ ^]^ dPCR offers enhanced specificity, precision, and tolerance to inhibitors, thereby finding indispensable applications in infectious diseases,^[^ ^4^, ^5^ ^]^ oncology,^[^ ^6^ ^]^ and noninvasive prenatal testing (NIPT),^[^ ^7^ ^]^ thus propelling the next‐generation technique in precise diagnostics.^[^ ^8^ ^]^

The potential of dPCR as the primary staple of future molecular diagnostics has led to tremendous efforts and breakthroughs in developing novel dPCR methods and platforms in the last two decades.^[^ ^3^, ^9^ ^]^ Advances in microfluidic large‐scale digital partitioning methods, including flow focusing,^[^ ^10^ ^]^ step emulsification,^[^ ^11^ ^]^ SlipChip,^[^ ^12^ ^]^ and soft‐lithographed valving,^[^ ^13^ ^]^ self‐digitization,^[^ ^14^ ^]^ or self‐priming micro‐chambers ^[^ ^15^, ^16^ ^]^ have enabled dedicated dPCR platforms with improved resolution, efficiency and reliability. However, these dPCR methods rely on microfabricated devices, specialized instruments, and complicated procedures compared to quantitative PCR (qPCR), limiting their standardization and implementation in clinical settings. Specifically, microfluidic dPCR chips encounter issues such as the nuances of chip handling, sub‐optimal sample utilization, and constrained throughput, particularly evident at the “world‐to‐chip” interface.^[^ ^17^ ^]^ Moreover, multiplexing in dPCR is essential because it helps transform biomarker discoveries from next‐generation sequencing (NGS) into affordable and routine molecular tests.^[^ ^18^, ^19^ ^]^ However, the multiplexity of dPCR is often constrained by factors such as limited spectrally distinct fluorophores, elevated fluorescence background and non‐specific amplification, and high probe costs when employing amplitude or color combination‐based strategies.^[^ ^20^ ^]^ Although melting curve analysis (MCA) is a powerful approach to interrogate complex sequence variants with clinical significance,^[^ ^21^ ^]^ its adaptation to dPCR necessitates continuous temperature adjustments and the acquisition of multicolor fluorescence intensities from numerous compartments,^[^ ^22^, ^23^ ^]^ posing challenges for droplet‐based dPCR systems or proving time‐consuming in microchamber‐based dPCR platforms. Given these challenges, a pronounced demand exists for a platform that not only tackles cost‐related concerns but also simplifies operations and enhances the multiplexing capabilities of dPCR. This demand can be addressed through the synergy of innovations in microfluidics, multiplexing chemistry, and advanced instrumentation—a convergence that promises to bolster the applicability and accessibility of dPCR across diverse clinical applications.

Here, we unveil the OsciDrop dPCR system, a revolutionary all‐in‐one, chip‐free dPCR platform, specifically engineered to transform high‐multiplex nucleic acid testing (NAT). This system extends our recently reported chip‐free droplet generation technique, OsciDrop,^[^ ^24^ ^]^ which allows pipette‐based printing of monodisperse droplets using innovative symmetrical oscillation and optimized pipette tip design. These enhancements ensure superior droplet uniformity and robustness, crucial for accurate dPCR analysis, and markedly diminish the system's sensitivity to operator errors, thus elevating its user‐friendliness and reliability for high‐throughput applications. Crucially, the OsciDrop system breaks new ground in cost‐effective consumables and high‐performance droplet generation, seamlessly integrating dPCR modules with improved fluid handling and machine learning‐based data processing. The efficacy of this system is demonstrated in its precision quantification of nucleic acid targets, as evidenced in multiplex dPCR assays targeting oncological markers. A standout feature of the OsciDrop system is its compatibility for high‐multiplex dPCR genotyping at scale, realized through our innovative multicolor digital stepwise melting analysis (dSMA),^[^ ^25^ ^]^ a technique offering unprecedented multiplexing capacity in dPCR. Overall, the OsciDrop dPCR system is a significant leap forward in molecular diagnostics, outperforming existing dPCR platforms in efficiency, multiplexing capability, cost‐effectiveness, and operational simplicity (Table S1, Supporting Information). Poised to broaden the adoption of dPCR in precision medicine, the OsciDrop system effectively addresses key challenges and heralds a new era in molecular diagnostics.

## 2 Results

### 2.1 Design and Principle of the OsciDrop dPCR System for High Multiplex Detection

**Figure 1:**
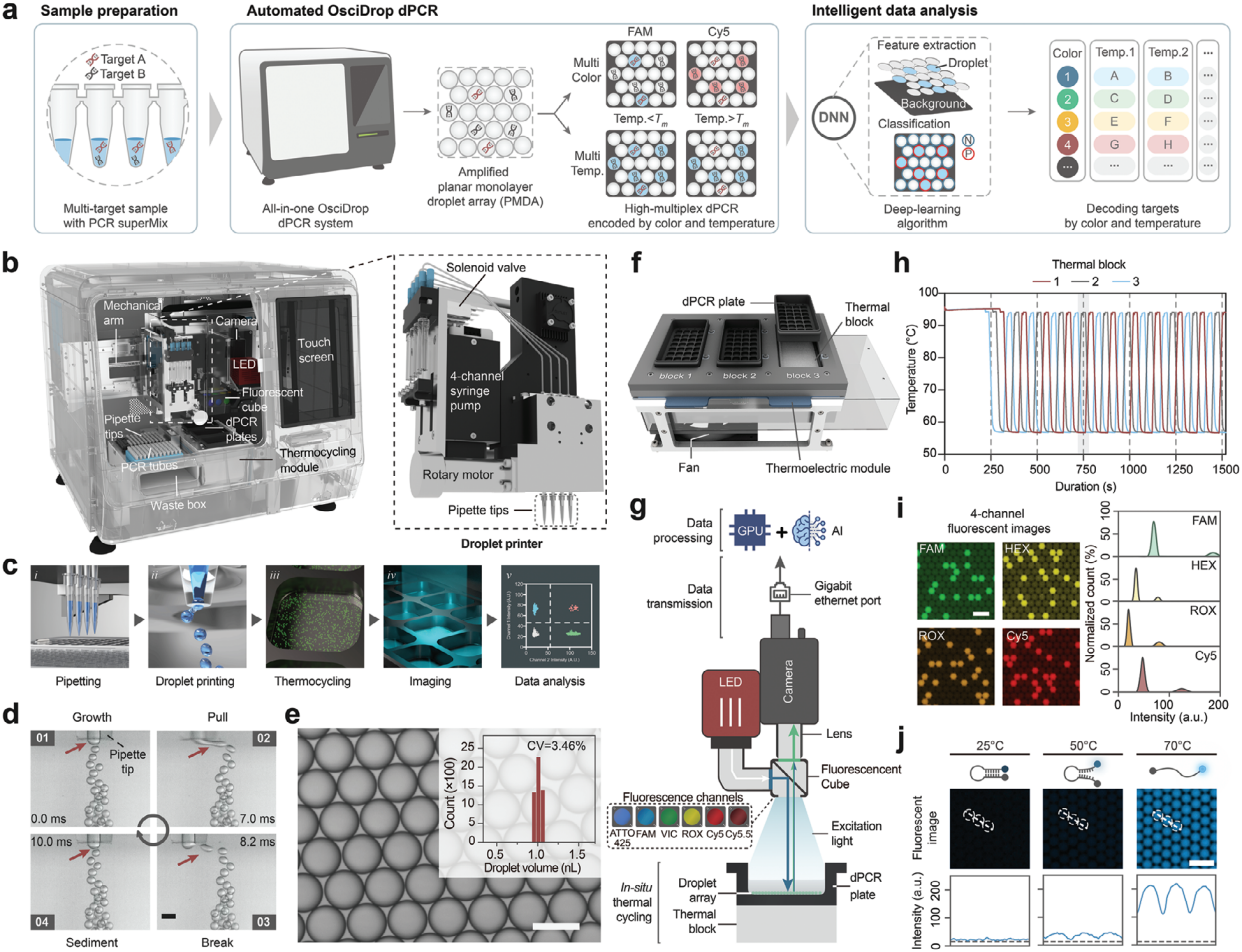
Overview of the All‐in‐One OsciDrop dPCR System. a) High multiplex dPCR using the OsciDrop system: Samples mixed with dPCR supermix undergo an automated process, covering liquid handling, dPCR amplification, multi‐temperature multicolor fluorescence imaging, and deep neural network (DNN) image analysis. b) The 3D illustration of the system with an enlarged view of the droplet printer highlights its compact and integrated design. c) The automated OsciDrop dPCR process. d) High‐speed imaging of oscillation‐based droplet generation. Scale bar: 200 µm. e) The histogram shows a bright‐field image of the planar monolayer droplet array (PMDA) with uniformity. Scale bar: 150 µm. f) The structure of the thermocycler. g) 6‐color fluorescence reader design. h) Thermocycling performance with red, gray, and blue lines representing thermal blocks 1, 2, and 3. i) Fluorescence images of the dPCR Starter kit with accompanying intensity histograms of droplets. Scale bar: 300 µm. j) Fluorescence imaging of a molecular beacon PMDA at 25, 50, and 70 °C with line‐scan plots, indicating the increase of droplet fluorescence intensities at higher temperatures compared with the background (the black dash line). Scale bar: 300 µm.

The OsciDrop dPCR system integrates automated processes for high‐throughput multiplex dPCR. Central to its design is the OsciDrop technique, which involves horizontally oscillating pipette tips within oil‐filled microwells to precisely control the volume and number of generated droplets. Its unique design synergizes the use of various components, such as robotic liquid handling, pipette‐based droplet printing, in situ thermocycling, and multicolor fluorescence imaging with intelligent data processing (**Figure** **1**a; Figure S1, Supporting Information). The system design streamlines the digital PCR workflow and utilizes cost‐effective consumables. A key innovation in this system is the precision control over droplet volume and number, achieved through a programmable oscillator and a 4‐channel high‐precision syringe pump. These components work in unison, producing uniform nanoliter droplets in dPCR plates, facilitated by disposable plastic pipette tips with an optimized orifice inner diameter of 160 µm (Figure 1b,c). This modification enhances manufacturability and precision, improving upon the previous 120 µm inner diameter.^[^ ^24^ ^]^ We evolved our previously introduced asymmetrical oscillation technique ^[^ ^24^ ^]^ to a refined symmetrical waveform with periodic stops (Table S2, Supporting Information). This modification significantly minimizes the required oscillation amplitude, mitigating lateral droplet displacement and disturbance (Figure 1d; Movie S1, Supporting Information). Once generated, droplets spontaneously settle at the center of the microwell. After the initial denaturation step, the droplets arrange themselves into planar monolayer droplet arrays (PMDAs) in the microwell, exhibiting remarkable stability during thermal cycling and dSMA analysis (Figure 1e; Movie S2, Supporting Information). In designing the pipette tips and dPCR plates, we meticulously adhered to the guidelines set forth by the Society for Laboratory Automation and Screening (SLAS, USA). Such adherence ensures the integrity of the experimental procedure and paves the way for efficient injection molding techniques, thereby facilitating cost‐effective mass manufacturing (Text S1, Supporting Information).

The dPCR system incorporated a high‐performance thermocycler comprising three flat thermal blocks, enabling simultaneous and independent thermocycling of three 32‐microwell dPCR plates (Figure 1f). An integrated fluorescence reader with a top‐down orthogonal optical pathway provided high illumination uniformity for in situ imaging. This reader is equipped with motorized filter sets, including six fluorescence channels, enabling color‐based multiplexing (Figure 1g; Figure S8, Supporting Information). The in situ thermocycler coordinated with the fluorescence reader, allowing 6‐color imaging at various temperatures to identify diverse targets with specific melting temperatures (*T*_m_) in a 2D encoding space of fluorescence probes and thermodynamic parameters (Figure 1a). This combinatory multiplexing strategy has been previously suggested but is not widely accessible among commercialized dPCR platforms.^[^ ^26^, ^27^ ^]^ Additionally, the thermocycler can perform three different melting analyses simultaneously, which increases flexibility and throughput and decreases the cost and turnaround time for multiplex dPCR assays.

### 2.2 Operation of the dPCR System

To perform dPCR experiments using the dPCR system, the samples, primers, probes, and dPCR Supermix were combined in 8‐strip PCR tubes and placed in the tube rack of the carrier. Following the setup of the dPCR reaction parameters such as droplet count, thermal cycling conditions, and imaging channels and exposure conditions (Movie S3, Supporting Information), the dPCR system performed the following automated steps (Figure 1c): 1) aspiration of samples into the array of pipette tips using positive oil displacement; 2) pipette droplet printing of PMDAs in the dPCR plates; 3) in situ thermocycling of the PMDAs; 4) capture of multicolor fluorescence images using the fluorescence reader; and 5) intelligent image processing by machine learning algorithms. The standard system setting can accomplish multiplex dPCR assays for 24 samples in three hours, with 20 000 droplets per sample partitioning, while significantly reducing hands‐on time to less than 5 min. The OsciDrop technology ensures precise control over droplet volume and quantity, offering flexibility in assay design.^[^ ^24^ ^]^ During the development of dPCR assays, we can also choose 10 000 or 5000 droplets per sample to expand the throughput to 48 or 96 samples per run and set three 32‐well dPCR plates with different thermocycling and imaging parameters, thus reducing the assay optimization cost and boosting the efficiency. Besides, the microwells containing amplified droplets can be individually retrieved for downstream analyses, such as amplicon sequencing.

### 2.3 Instrument Performance Characterization

To optimize the performance of the dPCR system, we systematically evaluated essential module functions, including droplet printing, thermocycling, and fluorescence detection. Liquid handling and droplet printing processes were optimized, increasing the partition utilization rate from ≈94% to >98% (Text S1, Figures S2 and S3, Supporting Information), which contributes to the expansion of the upper limit of the measurement (Figure S4, Supporting Information). By utilizing 4‐channel pipette tips, we achieved a printing throughput of 28 800 droplets per minute, generating 480 000 droplets in droplet microwells for 24 samples within 20 min. Notably, the droplet volume exhibited ultra‐low variability (1.010 ± 0.035 nL, CV = 3.460%, n = 5000) (Figure 1e; Figure S5, Supporting Information), ensuring accurate dPCR quantification.^[^ ^28^ ^]^ The planar monolayer droplet arrays (PMDAs) stability was demonstrated, with no observed cross‐contamination or amplicon leakage even after two weeks (Figure S6, Supporting Information).

The thermal blocks in the system exhibited ramping rates exceeding 2 °C s^−1^, ensuring uniform and efficient thermal cycling for up to three dPCR plates simultaneously (Figure 1h). The standard deviation across the heating area of the three blocks was within 1% when set at specific temperatures (Figure S7, Supporting Information). The thermal control of each block was independently confirmed, showcasing ramping rates of ≈3.0 °C s^−1^ for heating and 2.5 °C s^−1^ for cooling (Figure 1h; Figure S6b, Supporting Information). The fluorescence reader in the system effectively mitigated fluorescence spectral crosstalk, ensuring accurate detection of positive and negative droplets. Six fluorescence filters (ATTO‐425, FAM, VIC, ROX, Cy5, and Cy5.5) were optimized to minimize crosstalk, resulting in excellent linear relationships between concentrations and detected fluorescence intensities for droplets generated from serially diluted solutions of fluorescence dyes (31–500 nm) with R^2^ >0.99 (Figure S8, Supporting Information). The performance of multicolor fluorescence imaging was evaluated using the dPCR Starter kit for screening genetically modified organisms (GMOs). Droplets containing target templates exhibited significantly higher fluorescence intensities than negative droplets, enabling accurate enumeration and calculating the concentration of multiple DNA targets (Figure 1i; Figure S6, Supporting Information). Moreover, the dPCR system seamlessly integrates coordinated thermocycling and fluorescence imaging, enabling post‐PCR thermodynamics analysis utilizing structured DNA probes, such as molecular beacons, to enhance sensitivity, specificity, and multiplexing.^[^ ^21^, ^29^ ^]^ For instance, by imaging the PMDA with a molecular beacon targeting the *HER2* gene at various temperatures (25, 50, and 70 °C), we observed a progressive increase in fluorescence intensity, resulting in enhanced recognition of negative droplets from 78.54 ± 7.50% at 25 °C to 98.85 ± 0.56% at 50 °C and 98.89 ± 0.36% at 70 °C (Figure 1j; Figure S9 and Text S2, Supporting Information).

### 2.4 Intelligent Machine Learning‐Guided Image Analysis

**Figure 2:**
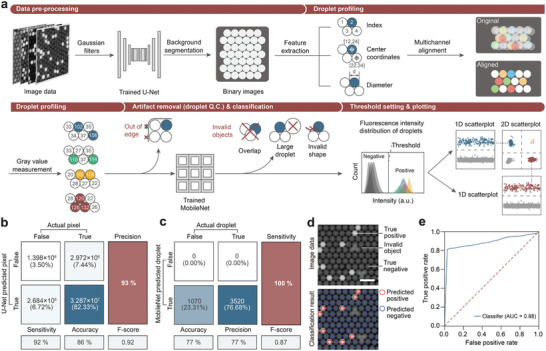
Machine learning‐guided droplet image analysis. a) Machine learning‐guided image analysis scheme: i) Image data refined with Gaussian filters, fed into U‐Net for droplet recognition. ii) The algorithm indexes droplets and acquires center coordinates and diameters. iii) Local droplet images were sent to the MobileNet classifier for quality control (Q.C.) to exclude invalid droplets and artifacts. iv) The intensity threshold setting and plotting of 1D and 2D scatter plots from valid droplet fluorescence intensities. b) Confusion matrix of the U‐Net identifier. c) Confusion matrix of the MobileNet droplet classifier. d) Fluorescence images of droplets subjected to deep learning analysis. Scale bar: 300 µm. e) ROC curve reflecting true/false positive rates across all processing steps and deep‐learning classifiers for droplet event prediction.

To enable intelligent image analysis in the OsciDrop dPCR system, we integrated supervised deep neural networks (DNNs) for droplet feature extraction and filtering within the software (**Figure** **2**a; Text S3, Supporting Information). Initially, the raw PMDA image data are denoised and fed into a trained U‐Net model ^[^ ^30^ ^]^ for droplet feature extraction, including center coordinate, diameter, and gray value (Figure S10a, Supporting Information). These features are then input into the MobileNet model ^[^ ^31^ ^]^ to filter artifacts, such as overlapping droplets, large droplets, and irregular features (Figure S10b, Supporting Information). The software calculates the frequency distribution of the droplets’ fluorescence intensity and determines the threshold for classifying positive and negative events.

Regarding the performance of individual modules, the U‐Net and MobileNet models achieved precisions of 93% and sensitivities of 100%, respectively (Figure 2b,c). Utilizing the U‐Net model, we accurately segmented most droplets from the PMDA images with a precision of nearly 100% (Figure 2d). The receiver operating characteristic (ROC) curve, based on the true‐positive and false‐positive rates, achieved an area under the curve (AUC) score of 0.88 (Figure 2e). Additionally, a specialized droplet alignment algorithm was implemented to counteract pixel shifts induced by using fluorescence filters of different wavelengths, thus improving the accuracy of multicolor fluorescence clustering (Figure 2c; Figure S11, Supporting Information). Notably, the imaging analysis runs concurrently with imaging and is completed as soon as imaging finishes. This deep‐learning algorithm offers rapid analysis speed, processing an image of 5000 droplets in ≈3.5 s.

### 2.5 dPCR Quantification Performance

To rigorously evaluate the accuracy and reliability of the OsciDrop dPCR system, we compared its performance against four leading commercial dPCR systems: QIAcuity (Qiagen), QX200 (Bio‐Rad), Naica (Stilla), and Absolute Q (ThermoFisher). This comparison utilized a reference material (RM) for copy number concentration (NIM‐RM4061) provided by the National Institute of Metrology, China (NIM). To ensure consistency across tests, we employed the same primers and probes for all platforms and adhered closely to the instructions provided by each platform and the guidelines accompanying the reference material to measure copy number concentration (**Table** **1**). The results revealed a consistent measured concentration across all five systems, highlighting dPCR's strength in accurately quantifying nucleic acids. Notably, the concentration measured by the OsciDrop dPCR system, 1.08 × 10^4^ copies µL^−1^ (CV = 1.48%, *n *= 15), closely matched the certified concentration provided by NIM. Furthermore, the OsciDrop system exhibited an impressive partition utilization rate, calculated as the ratio of the total number of analyzed partitions to the theoretical number of partitions, achieving an exceptional rate of 99.73%. Additionally, the OsciDrop dPCR system required only 3 min of hands‐on time, distinguishing it as the most operation‐friendly among the tested platforms.

**Table 1:** Quantification performance validation using NIM‐RM4061 plasmid reference material on five digital PCR platforms.

Methods	Chip‐based droplets		Microchambers		OsciDrop
dPCR platforms	QX 200	Naica	QIAcuity	Absolute Q	This work
Measured concentration (×10^4^ copies µL^−1^)	1.08 ± 0.24	1.25 ± 0.05	1.19 ± 0.29	1.04 ± 0.28	1.08 ± 0.01
CV of measured concentration (%)	2.37	4.29	2.43	2.68	1.48
Numbers of replicates	15	12	15	15	15
Reaction volume [µL]	20	25	40	10	25
Measured Partition number^a)^	15171 ± 1191	20960 ± 1888	25457 ± 18	20465 ± 10	20045 ± 103
Partition utilization rate [%]	75.86 ± 5.96	N.A.	97.92 ± 0.07	99.83 ± 0.05	99.73 ± 0.52
Hands‐on time (minutes)^b)^	20	12	10	12	3

**Notes**: The certified concentration of NIM‐RM4061 is 1.07 × 10^4^ copies µL^−1^ with an expanded uncertainty of 0.08 × 10^4^ copies µL^−1^ (*k* = 2).

^a)^ Mean with a standard deviation of 15 replicates.

^b)^ The hands‐on time was recorded based on 15 samples (12 samples for Naica) by the same experimental operator; Actual hands‐on time may vary depending on the operator and level of proficiency.

Furthermore, the feasibility of OsciDrop for accurately measuring nucleic acid targets was systematically assessed using specialized DNA and RNA RMs developed by NIM (Text S4, Supporting Information). We evaluated the quantification accuracy of target DNA fragments from the whole genome using an RM for human genomic DNA quantification (gDNA) (Figure S12, Supporting Information). We also conducted a precision evaluation for one‐step reverse transcription dPCR (RT‐dPCR) using an RM consisting of in vitro transcribed RNA from SARS‐CoV‐2 ^[^ ^32^ ^]^ (Figures S13 and S14, Supporting Information). Our measured concentrations and corresponding standard deviations (SD) were consistent with the certified concentrations, within the expanded uncertainties of these RMs. These results highlight the quantitative accuracy of the dPCR system, establishing its reliability for precise nucleic acid quantification.

### 2.6 Assessment of *HER2* Gene Status in Breast Cancer

**Figure 3:**
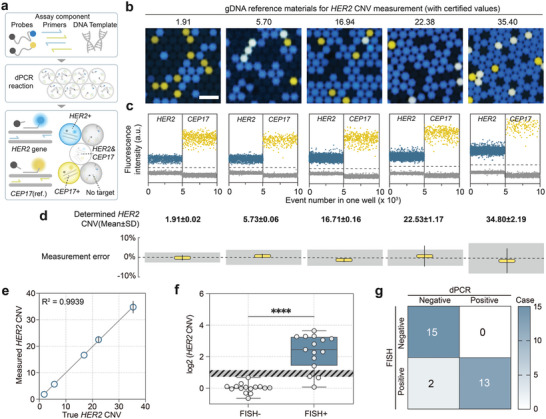
A duplex dPCR assay for *HER2* gene CNV assessment. a) Schematic illustration of duplex *HER2* CNV assessment assay. The precise CNV value can be determined by analyzing the number of droplets with the *HER2* gene and *CEP17* amplification. b,c) The count of *HER2*‐positive droplets increases with the rising CNV ratio in reference materials; Scale bar: 300 µm. d) A summary of the *HER2* CNV values determined from genomic DNA (gDNA) reference material derived from three independent experiments. The box plot presents the measured *HER2* CNV error, with the gray shadow denoting the expanded uncertainty of reference materials (for each group, *n* = 3, the error bars represent SD). e) Linear regression illustrating the correlation between true *HER2* CNV and measured *HER2* CNV of gDNA reference materials for *HER2* CNV measurement. Error bars represent SD f) Comparison between OsciDrop dPCR and FISH for qualitative detection of *HER2* overexpression using FFPE breast tissue samples. Box plot displaying determined *HER2* CNV by OsciDrop dPCR for FISH‐positive and negative clinical samples (*n* = 15, *t* = 7.704, *df *= 28, ^****^ indicates *p* <0.0001). g) The consistency matrix presents the count of matched cases between FISH and dPCR.

To evaluate the dPCR system's performance and practicality, we implemented a duplex *HER2* copy number variation (CNV) assay designed for breast cancer diagnostics (**Figure** **3**a). This assay precisely quantifies *HER2* copy number relative to *CEP17* (centromeric region of chromosome 17). With breast cancer now the most commonly diagnosed cancer worldwide,^[^ ^33^ ^]^ *HER2* gene over‐expression, observed in ≈15%‒30% of cases, become a critical target for *HER2*‐specific therapy.^[^ ^34^ ^]^ Currently, fluorescence in situ hybridization (FISH) is the standard for evaluating *HER2* over‐expression, involving complex manual procedures and subjective interpretation.^[^ ^35^ ^]^ To verify the accuracy and specificity of our assay, we used certified *HER2* copy number RMs from NIM (Figure 3b).^[^ ^36^ ^]^ Scatter plots display a clear separation between negative and positive clusters (Figure 3c), illustrating an increase in *HER2*‐positive droplets in reference materials with higher *HER2/CEP17* ratios. Measured values and standard deviations align within the expanded uncertainty of certified RMs and with an excellent linear relationship (Figure 3d,e), affirming the assay's capability for high‐precision quantification of CNV.

We then applied this assay to quantify *HER2* over‐expression in formalin fixation and paraffin embedding (FFPE) tissue samples from breast cancer patients, including 15 negative and 15 positive samples previously identified by FISH (Figure S15, Supporting Information). Using a *HER2/CEP17* ratio above 2.0 to indicate overexpression and below 1.5 for normal expression,^[^ ^37^ ^]^ our dPCR results matched 93.3% (28/30) of the FISH results (Figure 3f,g). In two cases where dPCR did not detect *HER2* amplification, FISH‐positive results were deemed due to amplified and aggregated *HER2* and *CEP17* signals.^[^ ^35^ ^]^ Although discrepancies between FISH and dPCR exist, it's important to recognize that clinical diagnosis often involves a combination of different tests and patient symptoms. Moreover, our findings align with previous chip‐based *HER2* dPCR assays,^[^ ^11^ ^]^ proving the high accuracy and efficiency of our dPCR system for quantitative *HER2* CNV assessment.

### 2.7 Measurements of *EGFR* Variant Allele Frequency (VAF)

Lung cancer is a leading cause of cancer‐related deaths worldwide,^[^ ^33^ ^]^ with a significant portion attributed to non‐small cell lung cancer (NSCLC).^[^ ^38^ ^]^ Many NSCLC cases exhibit alterations in the human epidermal growth factor receptor (*EGFR*) gene, which *EGFR* inhibitors can target.^[^ ^39^ ^]^ Precise determination of *EGFR* variant allele frequency (VAF) is vital for predicting treatment response and determining the metastatic stage of the disease.^[^ ^40^ ^]^ While NGS and qPCR are commonly used for this purpose, they face limitations in sensitivity and cost. dPCR offers a more sensitive and accurate alternative for direct quantification of VAFs without the need for calibrations, as is required in qPCR. However, existing dPCR platforms often face limitations in VAF profiling due to their costly instruments and consumables, complex operation, and limited variant coverage.^[^ ^41^ ^]^

**Figure 4:**
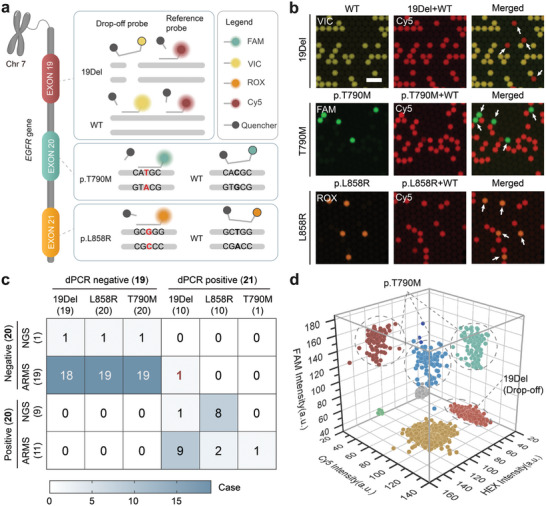
Quantifying *EGFR* variant allele frequency (VAF) by a quadruplex dPCR assay. a) Schematic illustration depicting the three *EGFR* target variants detected through the TaqMan‐based quadruplex dPCR assay. b) Separated and merged pseudo‐color fluorescence images illustrating 19Del, T790M, and L858R amplification; Scale bar: 300 µm. The red fluorescence originates from the total nucleic acid amplification. The yellow fluorescence indicates the wildtype of 19Del, and the green and orange fluorescence indicates the p.T790M and p.L858R, respectively. The white arrow above the merged image shows the droplets with mutant gene amplification. c) Correlation between negative and positive case numbers determined by dPCR, ARMS, and NGS. d) 3D scatter plot representing multicolor fluorescence intensity distribution of a clinical sample featuring a T790M and 19Del double‐locus variant. Yellow scatters denote 19Del wild type, while yellow and tomato red scatters (Cy5 positive) indicate wildtype T790M.

To address these challenges and facilitate timely clinical management of NSCLC, we developed a quadruplex dPCR assay using our dPCR system. This assay allows for the simultaneous evaluation of major *EGFR* VAF variants, including *EGFR* Exon 19 deletion (19Del), L858R, and T790M, in a single reaction (**Figure** **4**a). We evaluated the assay's sensitivity by analyzing gDNA spiked with variant templates at VAF levels of 0.1%, 1%, and 10%. The merged fluorescence images of planar monolayer droplet arrays (PMDAs) successfully distinguished different mutants across distinct fluorescence channels, achieving an excellent detection limit of 0.1% VAF for all tested *EGFR* variants (Figure 4b; Figure S16, Supporting Information).

To evaluate the clinical utility of the *EGFR* dPCR assay, we analyzed 40 FFPE tissue samples from NSCLC patients. We compared the results obtained by dPCR with those obtained using amplification refractory mutation system (ARMS) PCR or NGS in parallel (Table S4, Supporting Information). The dPCR results correlated well with those of ARMS (29/30) and NGS (10/10) (Figure 4c). Notably, our dPCR assay identified a positive case for the 19Del mutation that ARMS initially misdiagnosed. The droplets of this case were recovered from the microwell for amplicon sequencing, confirming as an uncommon C‐helix E 19Del mutant subtype with a 2.6% incidence rate in NSCLC ^[^ ^42^ ^]^ (Figure S17, Supporting Information). Furthermore, the dPCR assay demonstrated its capability by simultaneously detecting T790M and 19Del variants in a patient's FFPE sample (Figure 4d), underscoring its ability to allow multiplexed quantification of rare mutations at low copy numbers with enhanced precision and sensitivity.

### 2.8 High‐Multiplex *EGFR* Genotyping Using Multicolor dSMA

**Figure 5:**
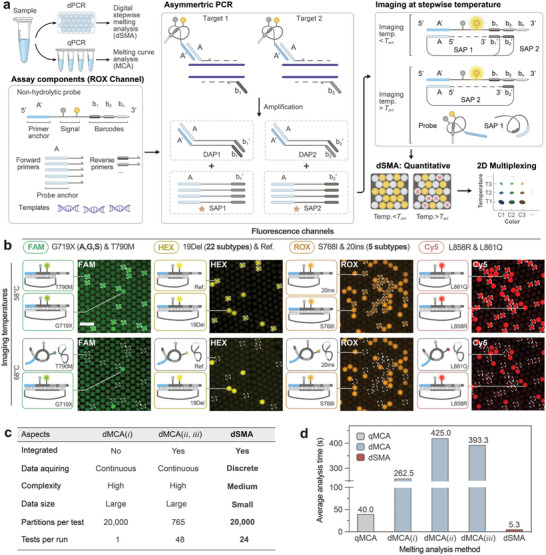
High‐multiplex *EGFR* genotyping using digital stepwise melting analysis (dSMA). a) The dSMA method, compatible with quantitative melting curve analysis (qMCA), utilizes non‐hydrolytic probes with a primer anchor at the 5′ end for forward primer binding, a quencher‐fluorophore pair, and barcodes at the 3′ ends for reverse primer binding. Forward and reverse primers are mixed in a 5:1 ratio. Excess single‐strain amplification products (SAPs) with probe anchors are generated during asymmetric PCR in droplets. These SAPs anneal to probes below their melting temperature (*T_m_*) and dissociate above *T*_m_, enabling temperature‐specific concentration determination and absolute quantification of various targets. Multiplexing is enhanced by using probes with distinct fluorophores. b) The probe status and fluorescent images of *EGFR* dSMA assay at 58 and 68 °C. Droplets containing SAP with lower *T*_m_ are circled by white dashed lines; Scale bar: 300 µm. c) Table outlining performance metrics of three melting analysis methods. d) The Bar chart compares the time taken by qMCA, dMCA (i,^[^ ^22^ ^]^ ii,^[^ ^43^ ^]^ and iii ^[^ ^44^ ^]^), and dSMA for one sample in a single fluorescence channel.

To further enhance the capability of dPCR for detecting more targets simultaneously, we developed the dSMA technique.^[^ ^25^ ^]^ The dSMA is rooted in the principle of combining fluorescent tagging and stepwise on‐off melting analysis of amplicon‐probe complexes to pinpoint distinct genetic variations. As depicted in **Figure** **5**a, we designed non‐hydrolytic probes with a reporter fluorophore and a quencher attached to the internal sequence. At the start (5′ end) of the probe, we designed a “primer anchor” (A′) for primer hybridization, while near the end (3′), a series of barcodes were designed for hybridization with single‐strand amplification products (SAPs). By adopting a 5:1 mix ratio, forward primers with “probe anchor” (A) and reverse primers with specific barcodes (b*_n_*, *n* represents the serial number of target) were combined into the assay to produce more SAPs during asymmetric PCR. After the amplification, SAPs were annealed to the probes by their probe anchors (A) at 3′ and target‐specific complementary sequences of b_n_ at 5′ (b_n_′), which increased the distance between the quencher and fluorophore to emit fluorescence. Then, we utilized stepwise temperature imaging on the same PMDA to acquire multiple image sets. Specifically for dPCR, the SAP detaches from the probe at temperatures above the SAPs' melting point, causing fluorescence quenching. This approach allowed us to analyze each set of images separately and calculate the copy numbers accurately to effectively identify and quantify various targets with distinct melting temperatures across different fluorescence channels. While this principle can also be applied to qualitative qPCR analysis using MCA, its application in dSMA within the OsciDrop dPCR system enables precise quantification of mutation rates, which is essential for companion diagnosis of cancers.

As an initial application, we developed a high‐multiplex *EGFR* assay for profiling major *EGFR* gene mutations in NSCLC, successfully distinguishing up to seven *EGFR* mutation variants spanning 35 subtypes with four probes and two melting temperatures (Figure 5b; Figure S18a–c, Supporting Information). Fluorescence images captured in four fluorescence colors at 58 and 68 °C revealed that all droplets containing mutations emitted fluorescence at 58 °C. In contrast, droplets containing mutations with melting temperatures of 68 °C were completely quenched at 68 °C (Figure 5b). Similarly, 1D scatter plots demonstrated a decrease in positive events at 68 °C compared to 58 °C for all four fluorescence channels (Figure S18d, Supporting Information). Consequently, we could distinguish and quantify all targets by imaging at only two temperatures (Table S5, Supporting Information), eliminating the need for continuous and intensive imaging at ramping temperatures. The precision of dSMA measurements was demonstrated through consecutive dilutions of target templates, with the linear regression curves exhibiting correlation coefficients (R^2^) above 0.90 (Figure S18e and Table S5, Supporting Information). Compared to melting curve analysis and existing melting‐analysis‐based multiplex dPCR approaches,^[^ ^22^, ^43^, ^44^ ^]^ our multicolor dSMA assay multiplies the number of genetic targets using discrete melting temperatures. This method eliminates the requirement for conducting a full melt curve analysis, reducing the time needed for each test. As a result, our approach lowers per‐test costs, enhances overall testing efficiency (Figure 5c,d), and improves specificity, which makes our dSMA assay a valuable tool for highly multiplexed profiling of clinically relevant mutant variants.

## 3 Discussion and Conclusion

The OsciDrop dPCR system described in this study introduces an automated and intelligent workflow for high‐multiplex genetic analysis, integrating instrumentation, consumables, reagents, and data management. The integrated instrumentation of our dPCR system confers significant advantages, such as cost‐effective consumables and compatibility with laboratory automation standards. The extension of the OsciDrop technique enables deterministic nanoliter droplet printing by positive oil displacement using disposable pipette tips, eliminates cross‐contamination from aerosols, and eliminates the need for expensive microfabricated devices. This advancement ensures cost‐effectiveness, scalability, and maximized sample utilization, which promote affordable and scalable dPCR assays. The automated nature of the system facilitates the rapid processing of many samples, leading to increased operational efficiency and a decrease in the chances of human error and contamination, which are common in manual methods. Consequently, the system enhances the reliability and reproducibility of the results obtained, as demonstrated by high precision in the quantification of nucleic acid reference materials.

Integrating in situ thermocycling and fluorescence imaging has expanded the system's multiplexing capacity, enabling broad quantitative genotyping with improved performance, better specificity, and reduced time consumption. Through fluorophore labeling and melting temperature co‐analysis, avenues have been opened for developing multiplexing approaches like color‐coded molecular beacons ^[^ ^21^ ^]^ and real‐time kinetics analysis,^[^ ^26^ ^]^ especially our newly‐developed dSMA technique for high‐multiplex genetic variants profiling.

Moving forward, our primary focus will be enhancing the OsciDrop dPCR system's workflow automation, process efficiency, and cost‐effectiveness for more streamlined nucleic acid quantification. Specifically, more effort will be devoted to allowing seamless integration of the OsciDrop dPCR system with automated sample preparation and assay setup. In particular, for the dSMA method, we are developing an assay with melting temperature‐dependent “ON‐OFF” switching to improve quantification accuracy for droplets containing multiple targets with the same fluorescence but different melting temperatures.^[^ ^25^ ^]^ Moreover, a focus area will be developing high‐multiplex dPCR assays for cancer liquid biopsy.^[^ ^45^ ^]^ Additionally, the compatibility of the OsciDrop system with various isothermal assays, such as digital loop‐mediated isothermal amplification (dLAMP) ^[^ ^46^, ^47^ ^]^ and digital CRISPR/Cas‐assisted assays,^[^ ^48^ ^]^ will be explored to enable rapid and early detection of infectious pathogens in critical care.

In conclusion, the OsciDrop dPCR system combines high throughput, cost‐effectiveness, and high multiplexity, which holds great promise as a next‐generation platform for rapid and quantitative molecular testing, paving the way for advancing personalized therapeutics and clinical diagnostics.

## 4 Experimental Section

### Reference Materials

Reference materials for calibration and validation purposes were obtained from NIM (Beijing, China). These materials included the reference material of human genomic DNA quantification (NIM‐RM4035‐2), the copy number concentration reference material for calibration of digital PCR instrument (NIM‐RM4061‐1, including primers and probes), the reference material for in vitro transcribed RNA of SARS‐CoV‐2 (GBW09298), and genomic DNA reference materials for *HER2* CNV measurement (GBW09116, GBW09117, GBW09118, GBW09119, GBW09120).

### Materials and Samples

Oligonucleotide primers, probes, and genes were synthesized by Sangon Biotech (Shanghai, China). All genes utilized in this experiment were synthesized and ligated into the pUC57 plasmid. The sequences of the oligonucleotides could be found in the (Tables S3 and S6, Supporting Information). The dPCR consumables, reagents, and kits were provided by Maccura Biotech (Chengdu, China). A customized dPCR Starter kit assessed instrument performance, detecting genetically modified organisms (GMOs) genes, including *T‐nos*, *P‐35S*, and *cp4‐epsps*, with *Lectin 4* as the internal reference gene. A 4‐color human gDNA quantification assay was developed to measure the gDNA reference materials from NIM. This assay detected *TGF‐β3*, *RNase P*, and *N‐HER2* in FAM (also Cy5), HEX, and ROX channels, respectively. DNA extraction and purification from FFPE samples were performed using Qiagen's GeneRead DNA FFPE Kit (180134, Qiagen, Germany). The *EGFR* gene VAF was quantified using the quadruplex *EGFR* Kit (GN7101342, Maccura). The high‐multiplex dSMA assay with reference materials for *EGFR* mutation screening was performed using a customized kit (GN7100342, Maccura).

### Experimental Setup and Procedures

The experiments were carried out using the OsciDrop dPCR system. The dPCR reaction mix in 8‐strip PCR tubes was prepared by combining 2× Supermix, 10× Primer & Probe, and varying volumes of templates, resulting in a final volume of 25 µL. Once the samples and consumables were ready, the automated dPCR process was initiated, which began with loading dPCR plates with 8 mL of droplet generation oil using an integrated piston pump. The droplet printer coordinated the 4‐channel syringe pump using 50‐µL glass syringes (30‐mm stroke, SETonic GmbH, Ilmenau, Germany) and a customized rotary motor with an oscillating frequency of 122 Hz to generate 20 000 1‐nL droplets for each sample (Text S1 and Figure S2, Supporting Information). Following the generation of droplets, the thermal blocks performed in situ PCR thermocycling. The precision of thermocycling was calibrated and evaluated using probe plates that accommodate fixed thermal sensors and an infrared camera to measure the dynamic temperature ramping rate and uniformity of thermal blocks. Subsequently, the dPCR system executed in situ multicolor fluorescence imaging at room or elevated temperatures to enable the digital melting analysis of multiple targets across each fluorescence channel. The imaging system was equipped with a motorized filter slider to accommodate up to six fluorescence filter sets.

### Bright‐Field Droplet Imaging and Droplet Volume Measurement

The droplets generated by the dPCR system were carefully pipetted to a flat‐bottom transparent polystyrene microwell and imaged using a Ti‐E inverted microscope (Nikon, Tokyo, Japan) equipped with a CoolSNAP HQ^2^ camera (Photometrics, Tucson, AZ, USA). The volume of 5000 droplets was measured originating from three independent experiments, with ten images (Figure S5, Supporting Information). The diameters of droplets were measured by a built‐in analysis module in NIS‐Elements software (Nikon).

### Time‐Lapse Imaging of Droplet Generation

To visualize the pipette droplet printing process from a side view, a cubic quartz cuvette (20 mm × 20 mm × 20 mm, Purshee Optical, Yixing, China) prefilled with 2‐mL droplet generation oil was utilized. Droplet printing was characterized by capturing time‐lapse bright‐field images at 3000 frames per second using a high‐speed camera (SC2, Edgertronic, San Jose, CA, USA).

### Multicolor Fluorescence Imaging

The detection limit and fluorescence crosstalks between channels were evaluated by imaging PMDAs containing fluorescence dyes at predefined concentrations. The fluorescence dyes, including ATTO425 (Sangon) 6‐FAM (HY‐66021, MedChemExpress, Dallas, TX, USA), 5(6)‐HEX (S26831, Yuanye Biotech, Shanghai, China), 5‐ROX (HY‐D0784, MedChemExpress), Cy5 (HY‐D0821, MedChemExpress), and Cy5.5 (Sangon), were diluted to 100 mm using DMSO (Macklin Biochemical, Shanghai, China) according to the provided instructions and stored at −80 °C. Series dilutions of each fluorescence dye were prepared using 2× dPCR Supermix to final concentrations of 500, 375, 250, 188, 125, 63, and 31 nm. Droplets were generated in dPCR plates, and 6‐color fluorescence imaging was performed following a 95 °C heating step to facilitate the self‐assembly of PMDAs in microwells.

### Image Processing and Statistic Analysis

The OsciDrop Analysis Pro software processed the original fluorescence images of dPCR experiments, calculated the target gene concentrations, and visualized the results using 1D and 2D scatter plots. The results were presented as mean ± SD (n = three or more independent experiments unless otherwise stated). The software allowed visualization of the results as 1D or 2D scatter plots and exportation of results as CSV sheets or original fluorescence images as TIFF files. Statistical analysis and visualization were performed in Origin Pro (OriginLab, Northampton, MA, USA).

### Quantification Performance Comparison

The certified concentration of NIM‐RM4061 plasmid reference material was characterized by isotope dilution mass spectrometry (ID‐MS) and droplet dPCR by QX200 (Bio‐Rad, Hercules, CA, USA), as stated by the NIM. All the assay components were designed and prepared following the instructions of the reaction kits from the manufacturers. For dPCR on the OsciDrop dPCR system and the Naica system (Stilla Technologies, Villejuif, France), the assays were prepared in 25‐µL reactions. For QX200, the assay was performed in 20‐µL reactions. For QIAcuity (Qiagen, Hilden, Germany), the assay was performed in 40‐µL reactions. For Absolute Q (ThermoFisher Scientific, Waltham, MA, USA), the assay was performed in 10‐µL reactions. The thermocycling conditions and fluorescence imaging parameters are listed in Table S7 (Supporting Information).

### *HER2* CNV Quantification

The *HER2* CNV quantification was performed in a 25 µL reaction containing 12.5 µL 2× dPCR Supermix, 0.6 µL DNA polymerase, 6.9 µL DNase‐Free Water, 2.5 µL 10× Primer & Probe, and 2.5 µL template. The working concentrations of the primer and probe were 400 and 200 nm, respectively. Each sample was tested in triplicate. For FFPE samples, Paraffin was removed from the 10‐µm slice. Qbit 4.0 (ThermoFisher) was used to measure the DNA concentration and diluted it to 10 ng µL^−1^.

### *EGFR* VAF Quantification

The synthesized plasmids were quantified and mixed with gDNA reference materials at concentration ratios of 0.1%, 1%, and 10%. The *EGFR* 19Del, L858R, T790M VAF quantification assay was performed in a 25 µL reaction, which contained 12.5 µL 2× dPCR Supermix, 0.6 µL DNA polymerase, 6.9 µL DNase‐Free Water, 2.5 µL 10× Primer & Probe, and 2.5 µL template. The DNA samples were extracted from clinical FFPE tissues, and their concentrations were measured using Qbit and diluted to 10 ng µL^−1^.

### dSMA *EGFR* Variants Profiling

The dSMA *EGFR* variants profiling was performed in a 25 µL reaction containing 12.5 µL 2× dPCR Supermix, 0.6 µL DNA polymerase, 4.4 µL DNase‐Free Water, 2.5 µL 10× Primer and Probe, and 5.0 µL template. For sample preparation, the T790M and G719X reference materials were mixed at the concentration ratio of 8:1, the reference gene plus 19Del, 20ins plus S768I, and L861Q plus L858R were all mixed at the concentration ratio of 3:1, stored at a concentration of 0.1 µg µL^−1^, and diluted to the average concentration of 10 ng µL^−1^ using TE‐buffer (T1120, Solarbio, Beijing, China) for reaction. Each sample was tested in three independent experiments.

### Multicolor dPCR Performance Characterization

The dPCR Starter kit (Maccura) detected genetically modified soybeans. The target GMO genes included *T‐nos*, *P‐35S*, and *cp4‐epsps*, with *Lectin 4* as the internal reference gene. The assay was performed using plasmid templates at concentrations of 3 × 10^4^ and 100 copies µL^−1^ along with no template control (NTC). Fluorescence images were exported and analyzed using ImageJ (NIH, USA) to obtain the fluorescence line‐scan profiles.

### Ethical Statement

Formalin‐fixed paraffin‐embedded (FFPE) samples from 30 patients with breast cancer and FISH diagnosis results were provided by Shanghai Ninth People's Hospital. Peking University Peoples Hospital provided nucleic acid samples extracted from 40 lung cancer tissues from patients. The Declaration of Helsinki conducted this study, and the protocol used to collect human tissue was approved by the Ethics Committee of Shanghai Ninth People's Hospital (DI: 023‐KY‐12) and Peking University People's Hospital (DI: 2023PHB206‐001). All patients provided written informed consent before the study. Personal identity information was hidden to protect individual privacy.

## Conflict of Interest

The authors declare no conflict of interest.

## Author Contributions

C.L., X.W., Y.H., J.F., L.M., and Y.Z. designed and performed the experiment, analyzed data, and performed data visualization. S.Y. and Y.F.L. gave conceptual advice and assisted in data analysis and visualization. W.H. evaluated and optimized the machine learning model. N.K., C.W., and D.S. provided the clinical samples with diagnostic results. Y.L. provided suggestions to improve the experiment design. L.D. and W.D. designed and supervised the project.

## Supporting information

Supporting Information

Supplemental Movie 1

Supplemental Movie 2

Supplemental Movie 3

## Data Availability

The data that support the findings of this study are available from the corresponding author upon reasonable request.
