# Supplementary material for: All‐In‐One OsciDrop Digital PCR System for Automated and Highly Multiplexed Molecular Diagnostics
Source: Adv Sci (Weinh). 2024 Mar 22;11(21):2309557. doi: 10.1002/advs.202309557 (PMC11151056; doi:10.1002/advs.202309557)
Supplement: Supplementary file 1 — Supporting Information [file ADVS-11-2309557-s004.pdf]

ADVANCED SCIENCE
Open Access

Supporting Information

for *Adv. Sci.*, DOI 10.1002/advs.202309557

All-In-One OsciDrop Digital PCR System for Automated and Highly Multiplexed Molecular Diagnostics

Caiming Li, Nan Kang, Shun Ye, Weihang Huang, Xia Wang, Cheng Wang, Yuchen Li, Yan-Fei Liu, Ying Lan, Liang Ma, Yuhang Zhao, Yong Han, Jun Fu, Danhua Shen, Lianhua Dong and Wenbin Du**

Supporting Information

All-in-One OsciDrop Digital PCR System for Automated and Highly Multiplexed Molecular Diagnostics

Caiming Li, Nan Kang, Shun Ye, Weihang Huang, Xia Wang, Cheng Wang, Yuchen Li, Yan-Fei Liu, Ying Lan, Liang Ma, Yuhang Zhao, Yong Han, Jun Fu, Danhua Shen, Lianhua Dong, Wenbin Du**

This file includes

Figure S1–S18

Tables S1–S7

Legend to Movie S1–S3.

Text S1–S4.

Supporting References.

Supporting Figures

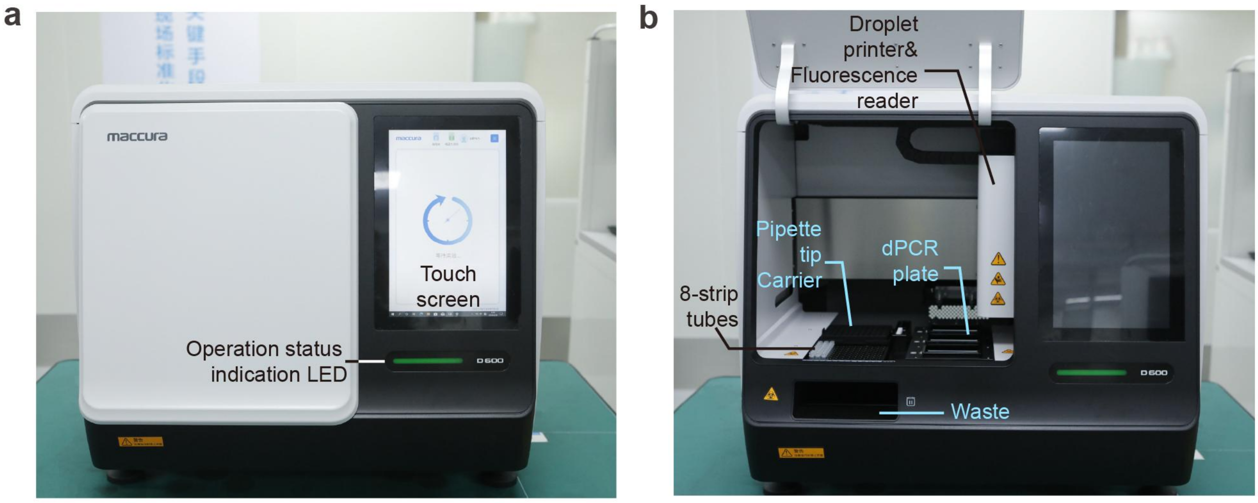

Figure S1. Images of the integrated OsciDrop dPCR system. External view (a) and internal view (b) of the OsciDrop dPCR system.

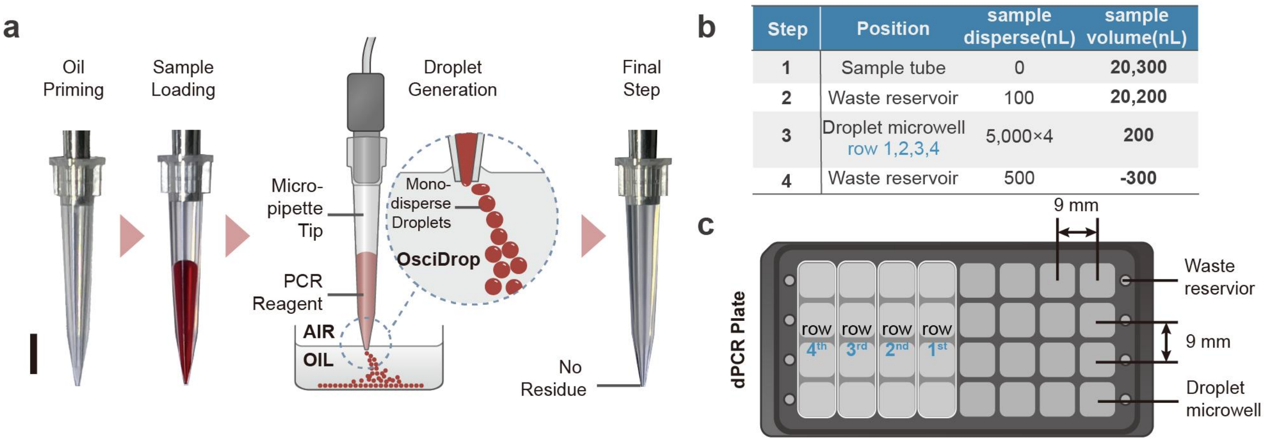

Step	Position	sample disperse(nL)	sample volume(nL)
1	Sample tube	0	20,300
2	Waste reservoir	100	20,200
3	Droplet microwell row 1,2,3,4	5,000×4	200
4	Waste reservoir	500	-300

Figure S2. Optimized OsciDrop droplet generation procedure. (a) Diagrams illustrating the liquid handling and droplet printing process. The OsciDrop technique was employed to split all red ink solutions into monodisperse droplets; Scale bar: 3 mm. (b) The droplet generation process was refined to maximize sample usage. The table presents sample processing stages, pipette tip positions, and dispensed sample volumes. (c) Top-down schematic view of the dPCR plate, indicating the sequential process positions.

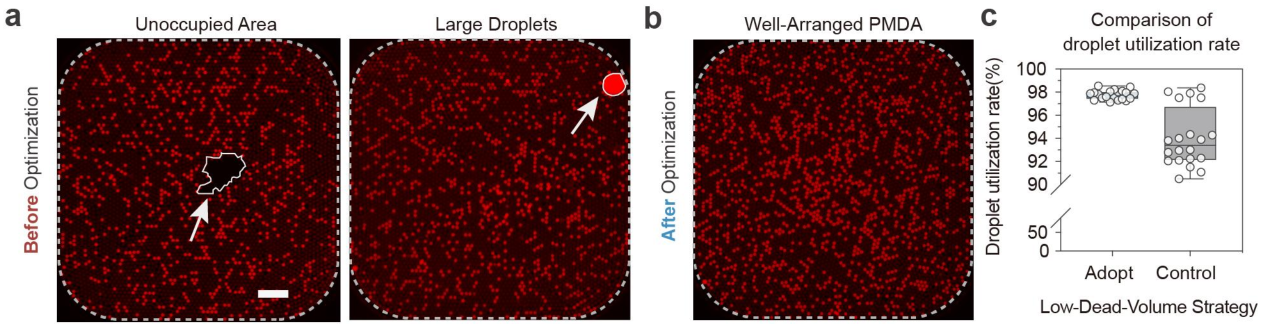

Figure S3. Enhanced PMDA assembly through optimized droplet printing procedure. (a) Before optimization, some PMDAs exhibit defects, including unoccupied areas or large droplets. Scale bar: 1 mm. (b) Post low-dead-volume optimization, nearly flawless PMDAs were achieved. (c) The droplet utilization rate for the amplified dPCR Starter kit assay improved by adopting low-dead-volume optimization, showing reduced instances of overlapped droplets, large droplets, and unoccupied microwell areas.

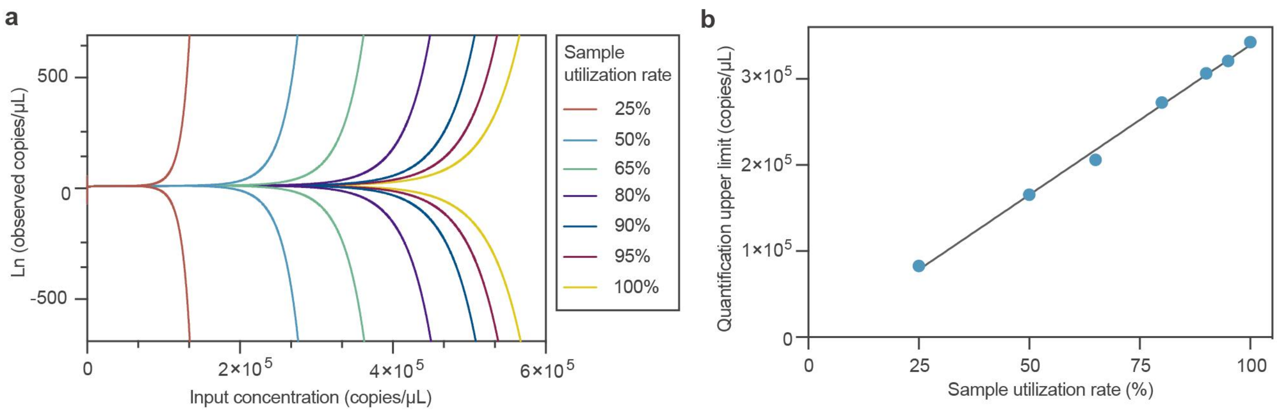

Figure S4. Theoretical calculation of quantification upper limit based on Poisson distribution. (a) Line plot depicting the 95% confidence interval (CI) of various sample utilization rates based on theoretical calculation. (b) A linear regression plot illustrates the correlation between sample utilization rate and quantification upper limit.

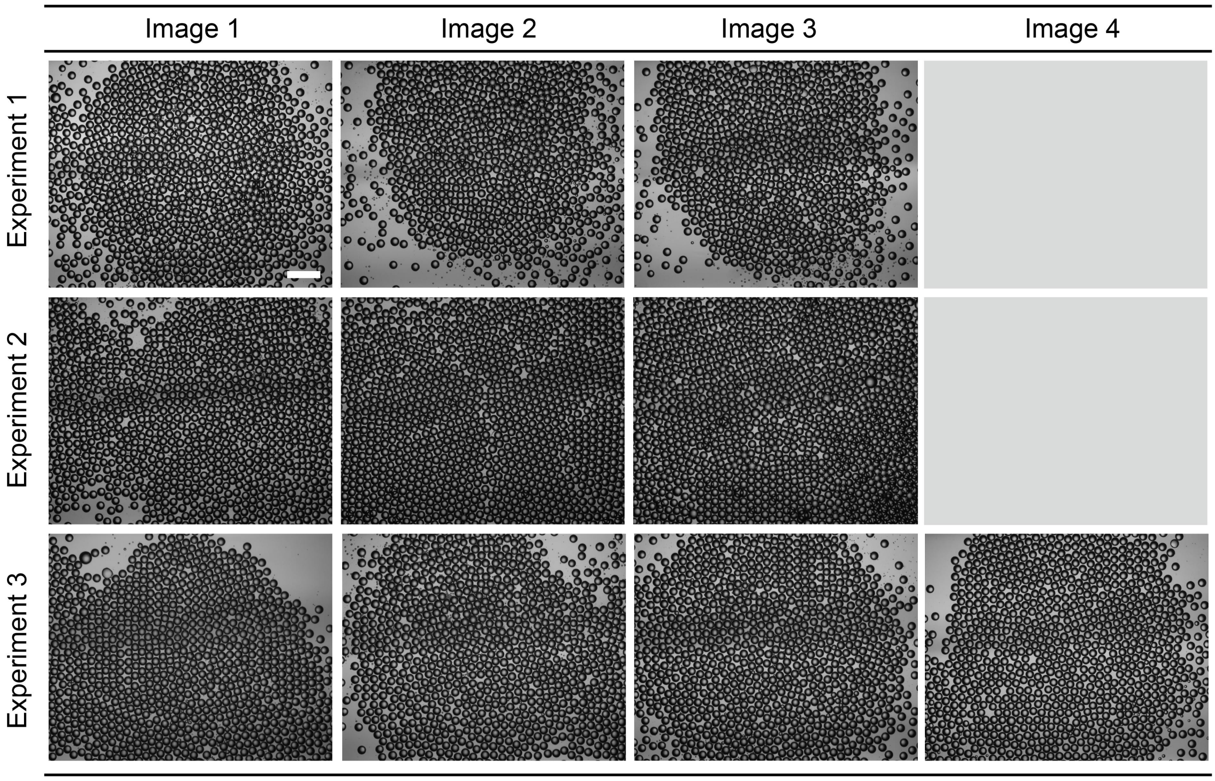

Figure S5. Serial images of generated 1-nL droplets. In a bright-field image of 1-nL droplets from three experiments, 500 droplets from each image are measured, and the mean volume of these generated droplets is calculated and introduced in Figure 1e. Scale bar: 500 μm.

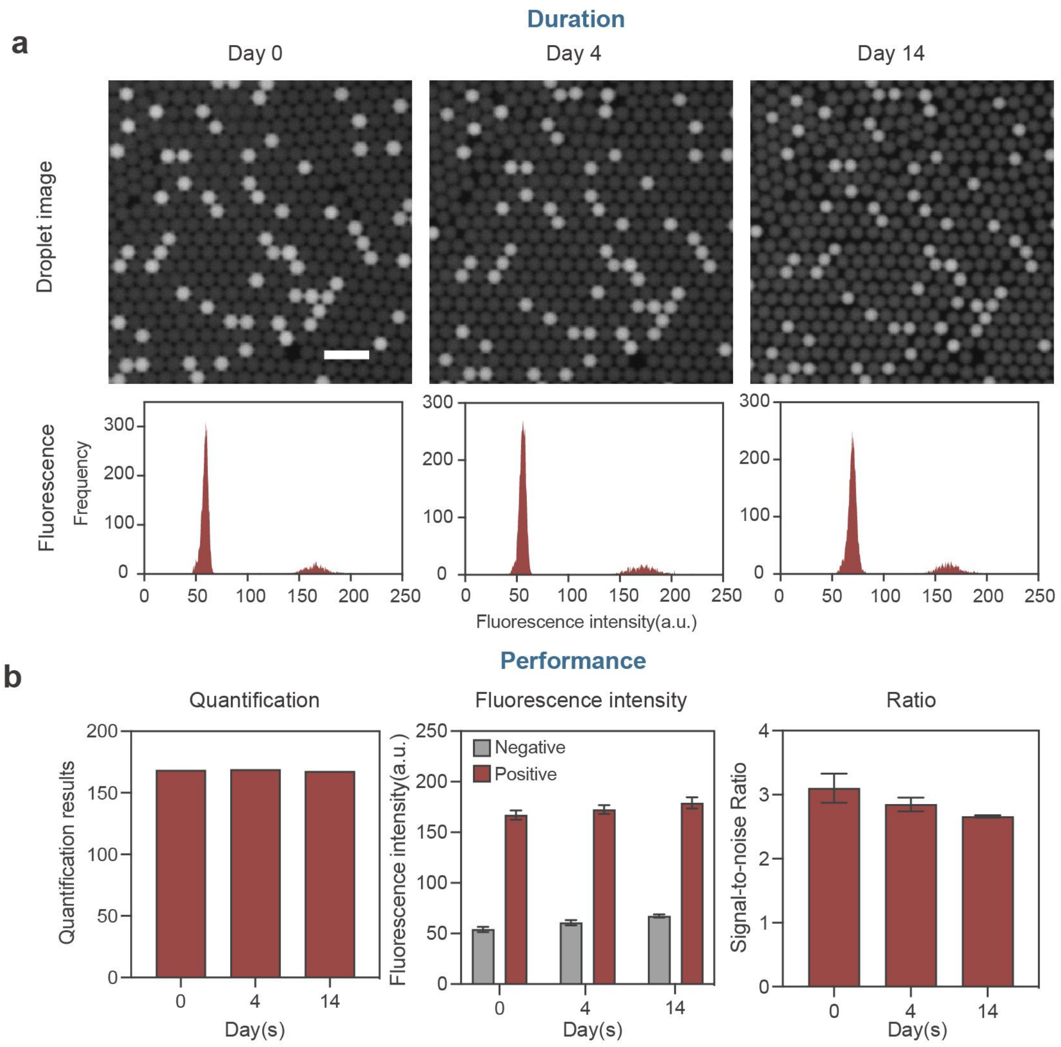

Figure S6. Enduring stability of PMDAs over time. (a) Fluorescence images of amplified PMDAs (dPCR Starter kit) after 0, 4, and 14 days, along with corresponding histograms of fluorescent intensity distributions. Scale bar: 400 µm. (b) Quantification, fluorescence intensity, and signal-to-noise ratio of PDMAs during extended storage. The signal-to-noise ratio decreased from 3.1 ± 0.23 to 2.7 ± 0.02 after two weeks due to minor droplet volume shrinkage. However, quantification results remained consistent, as positive and negative events were separated distinctly. Error bars represent standard deviations (n = 3).

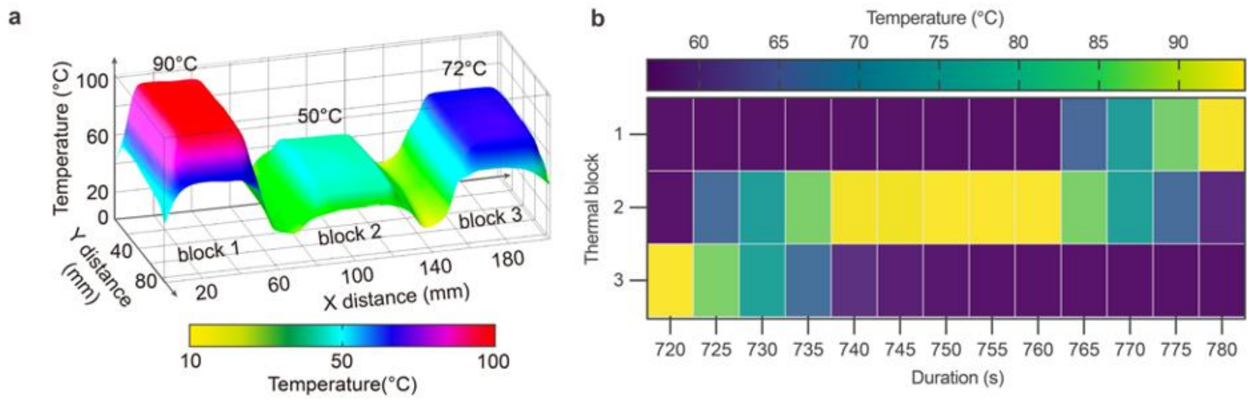

Figure S7. Validation of performance for the three thermal blocks in the thermocycler. (a) Independent temperature control was demonstrated across three thermal blocks, visualized using an infrared camera. (b) Temperature variations across the three blocks over 60 seconds.

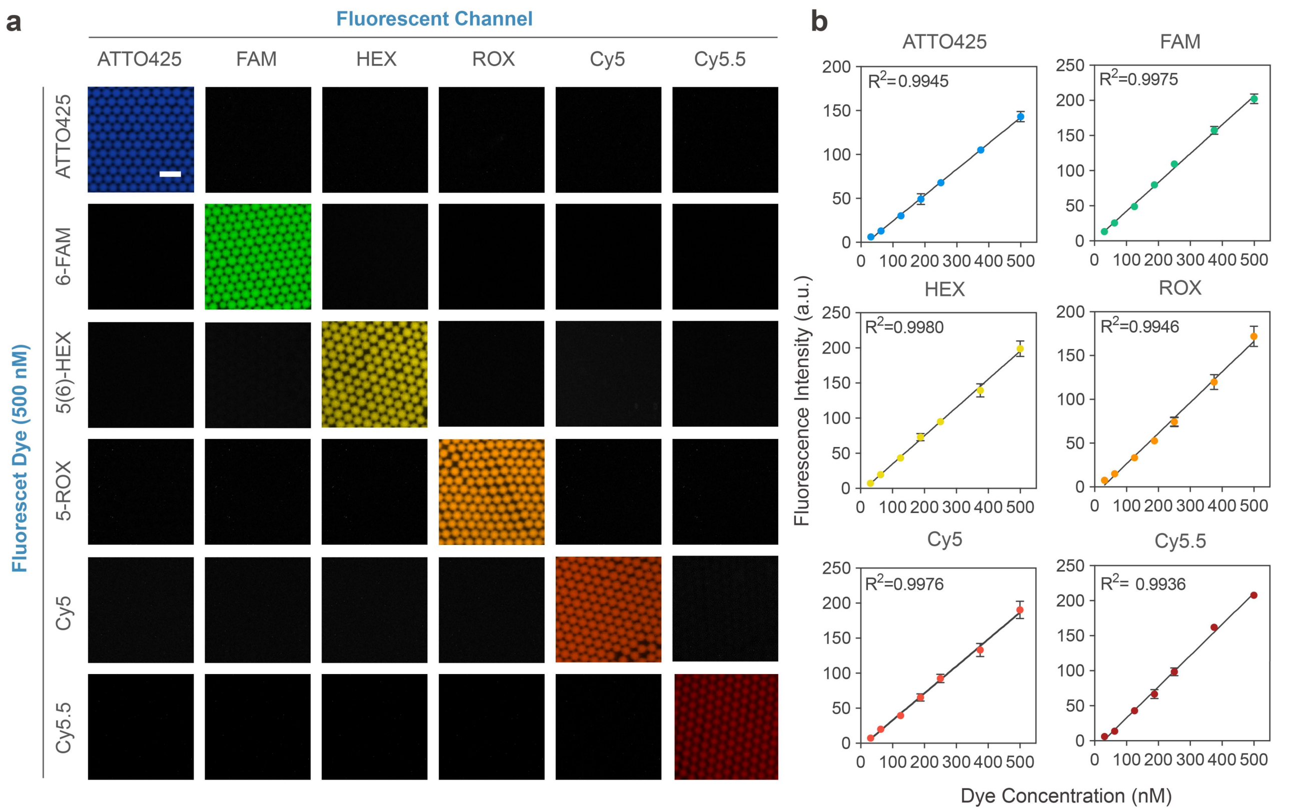

Figure S8. Validation of performance for the 6-color fluorescence reader. (a) Fluorescence imaging with minimal crosstalk between fluorescent channels and no fluorophore leakage was observed. Fluorescent images of dye droplets captured in six channels, portrayed in pseudo-colors. Scale bar: 500 µm (b) Serial dilution of fluorescent dye with corresponding measured fluorescence intensity linked to dye concentrations ranging from 500 nM to 31 nM ($R^2 > 0.99$). Error bars denote standard deviations from three repeats.

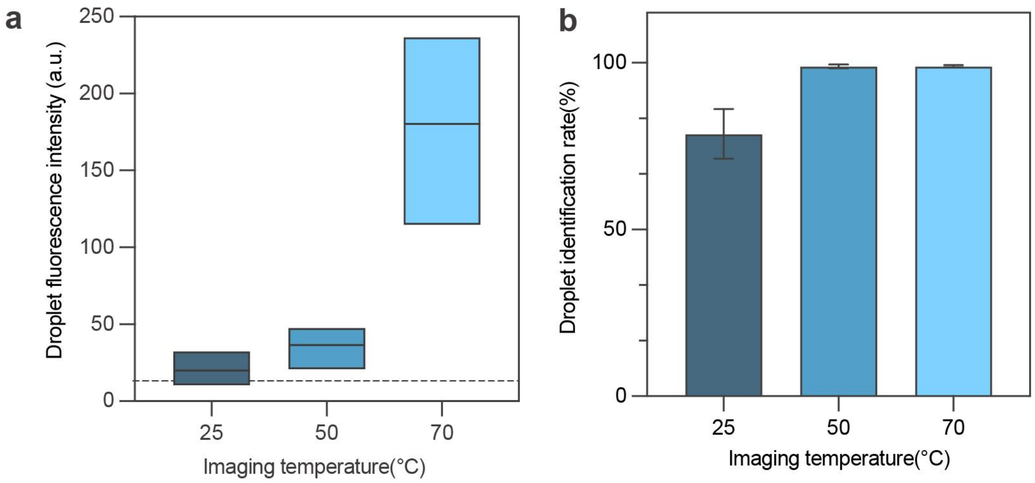

Figure S9. Post-PCR thermodynamics analysis of molecular beacon-based dPCR assays. (a) Box plot displaying grayscale values extracted from molecular beacon droplets imaging at 25 °C, 50 °C, and 70 °C. The dashed line represents the background fluorescent intensity. (b) Comparison of droplet identification rates for molecular beacon droplets at 25 °C, 50 °C, and 70 °C. The error bar indicates standard deviations (n = 5).

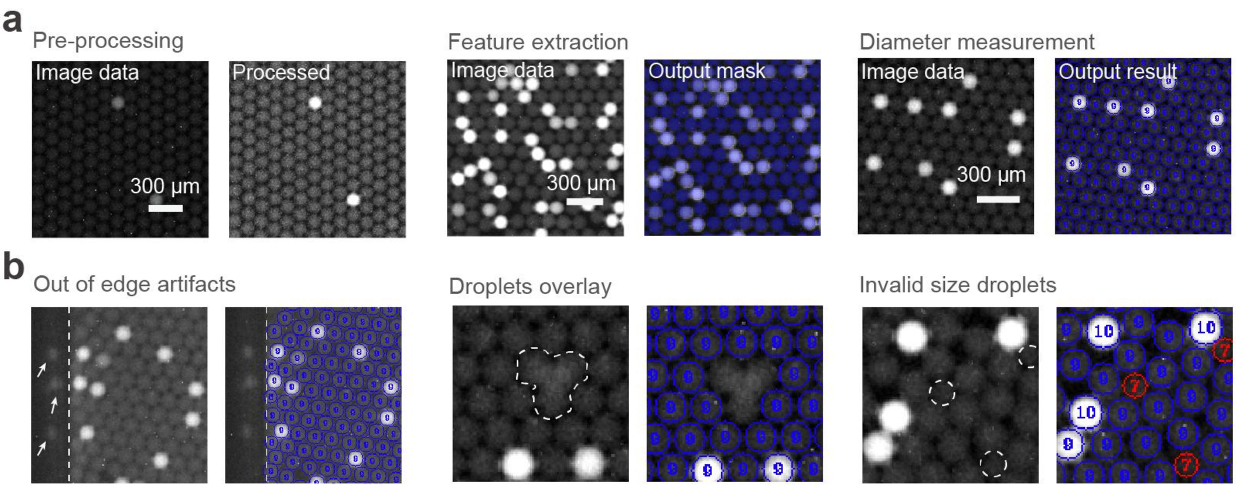

Figure S10. Image data process, feature extraction, and artifact removal. (a) The image data undergo denoising before being fed into the U-Net model for feature extraction and diameter measurement. (b) Three primary categories of droplet image artifacts: artifacts beyond the edge, multi-layer droplets, and improperly sized droplets. Arrows or dashed lines encircle the artifacts to indicate their presence.

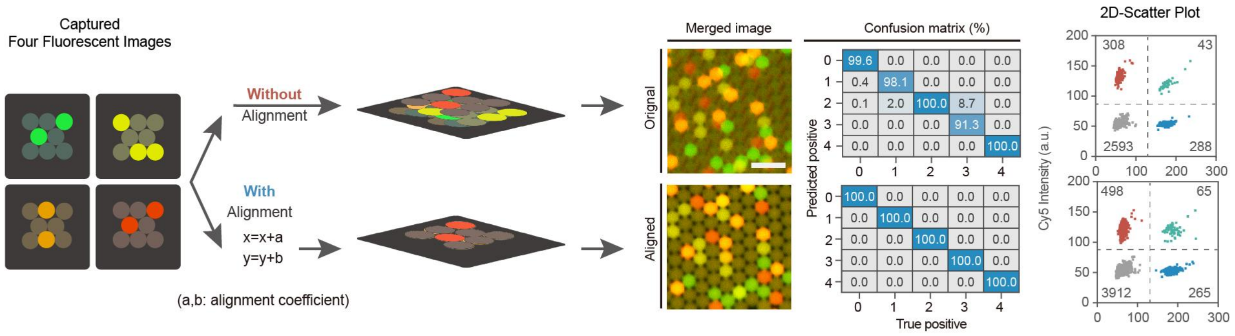

Figure S11. Alignment of Four-Channel Fluorescence Images of Droplet Arrays. Cartoon schematic illustrating the alignment process of four fluorescent channels. Comparison between the original and aligned images, generated by merging four fluorescence images from the dPCR Starter kit assay. The confusion matrix underscores the concordance between the actual count of single-positive, double-positive, triple-positive, and quadruple-positive droplets in each droplet and the anticipated count before and after alignment; Scale bar: 300 µm. The two-dimensional scatterplot showcases the identified droplet counts of the four clusters before and after alignment.

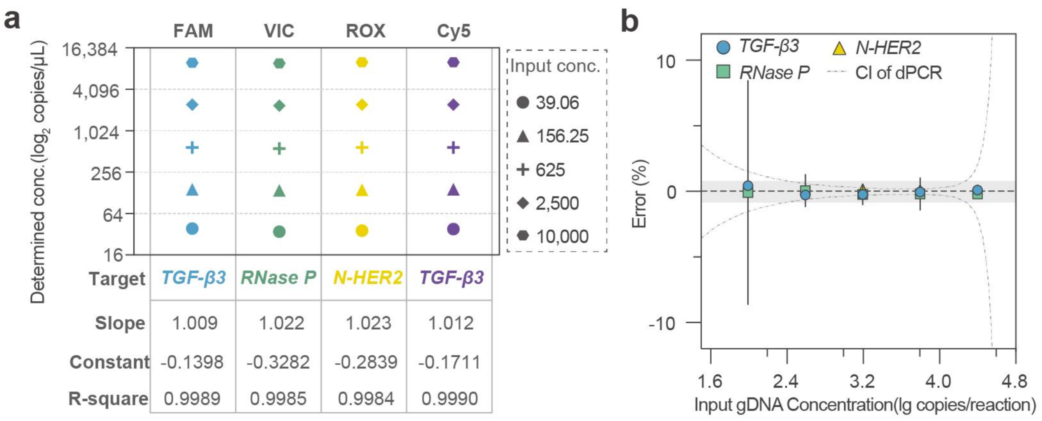

Figure S12. Validation of quantification performance using human genomic DNA (gDNA) standard reference materials (RMs). (a) Visualization and statistical representation of a serial dilution of human gDNA RMs utilized in the quantification assay. (b) The scatter plot displays the quantification error (%) with the expanded uncertainty indicated by the gray shade, and the dashed curves represent the 95% confidence interval (CI). Error bars represent the standard deviations of three replicates.

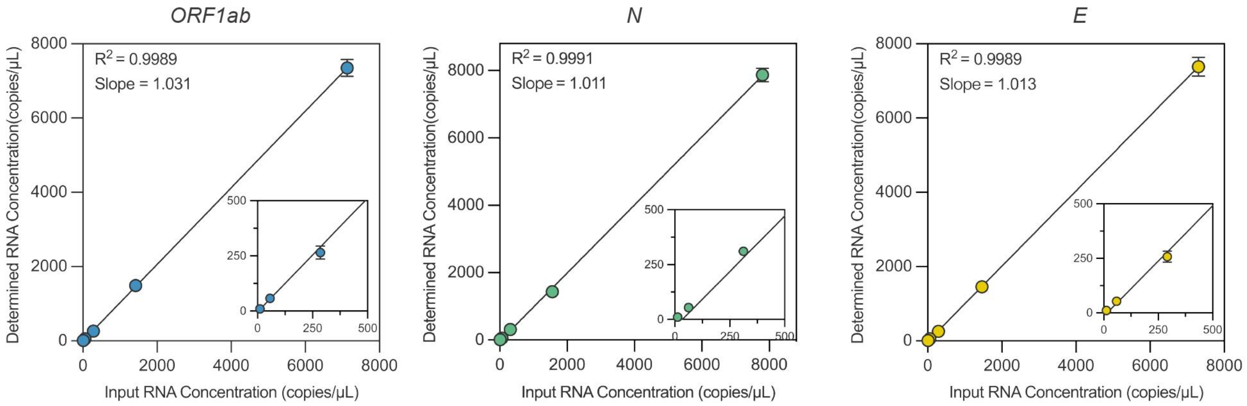

Figure S13. Linear Regression Analysis of SARS-CoV-2 RNA Reference Materials (RMs). Regression fit of results derived from the calculated concentration of *ORF1ab*, *N*, and *E* genes in the final template solution obtained via RT-dPCR using the OsciDrop dPCR system with serially diluted SARS-CoV-2 RNA RMs. The error bars represent standard deviations (n = 3). The concentration range (0 ~ 500 copies/μL) is shown in enlarged insets for clarity.

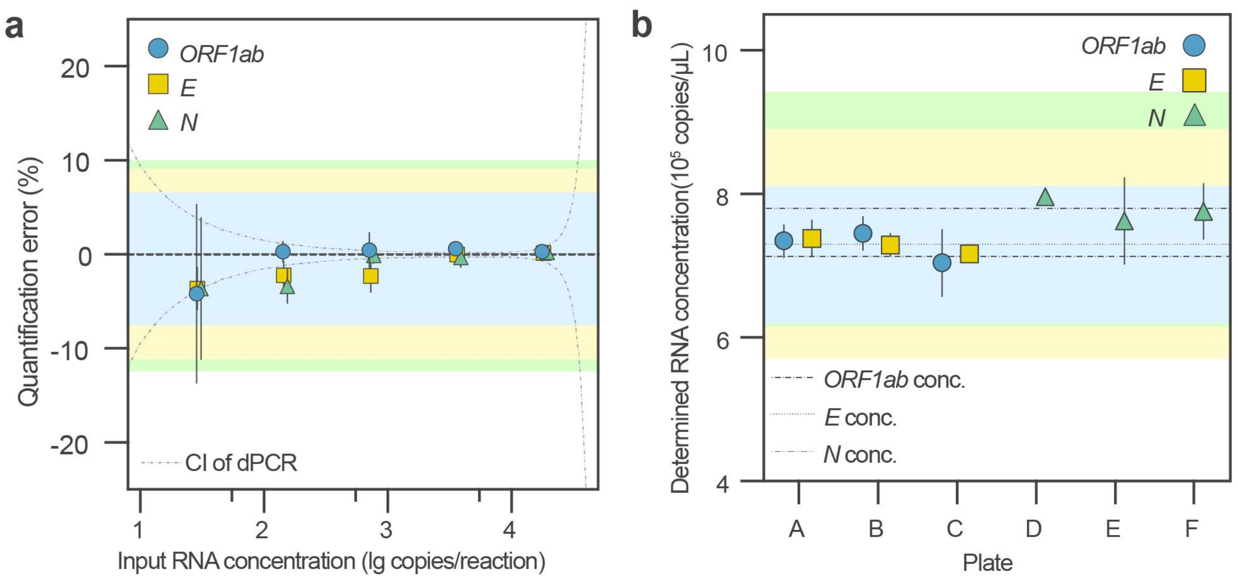

Figure S14. Validation of Quantification Performance Using SARS-CoV-2 RNA Reference Materials (RMs). (a) Scatterplot illustrating the quantification error (%) of SARS-CoV-2 RNA RMs. The circle, square, and triangle symbols represent *ORF1ab*, *E*, and *N* genes, respectively. Error bars denote standard deviations (n = 3). Blue, yellow, and green shadows signify the extended uncertainty (provided by RMs' certificate) for *ORF1ab*, *E*, and *N* genes. The dashed curve represents the 95% confidence interval (CI). (b) Scatterplot depicting dPCR quantification of SARS-CoV-2 RNA RMs in three plates. The circle, square, and triangle symbols represent *ORF1ab*, *E*, and *N* genes. Error bars represent standard deviations (n = 8). Blue, yellow, and green shadows indicate the expanded uncertainty (based on RMs' certificate) for *ORF1ab*, *E*, and *N* genes. Three types of dashed lines indicate the true concentrations of the target *ORF1ab*, *E*, and *N* genes provided by RMs.

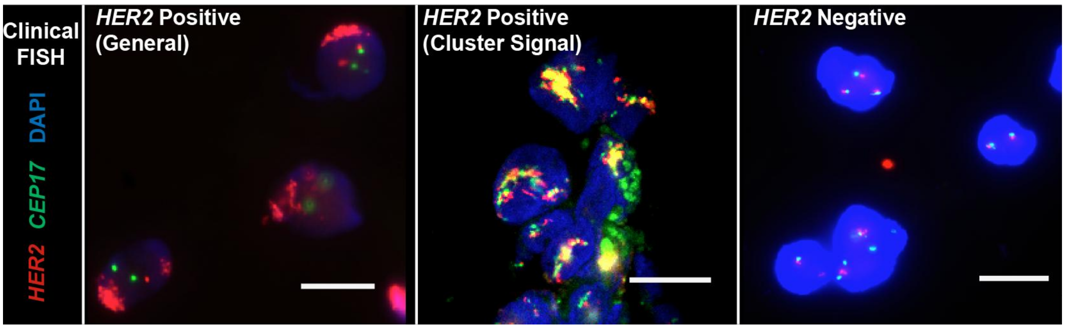

Figure S15. Representative FISH Results of Clinical Samples. *HER2* FISH results are displayed. Red, green, and blue fluorescence signify *HER2* gene amplification, *CEP17* gene amplification, and cell nuclei, respectively. Scale bar: 10 µm.

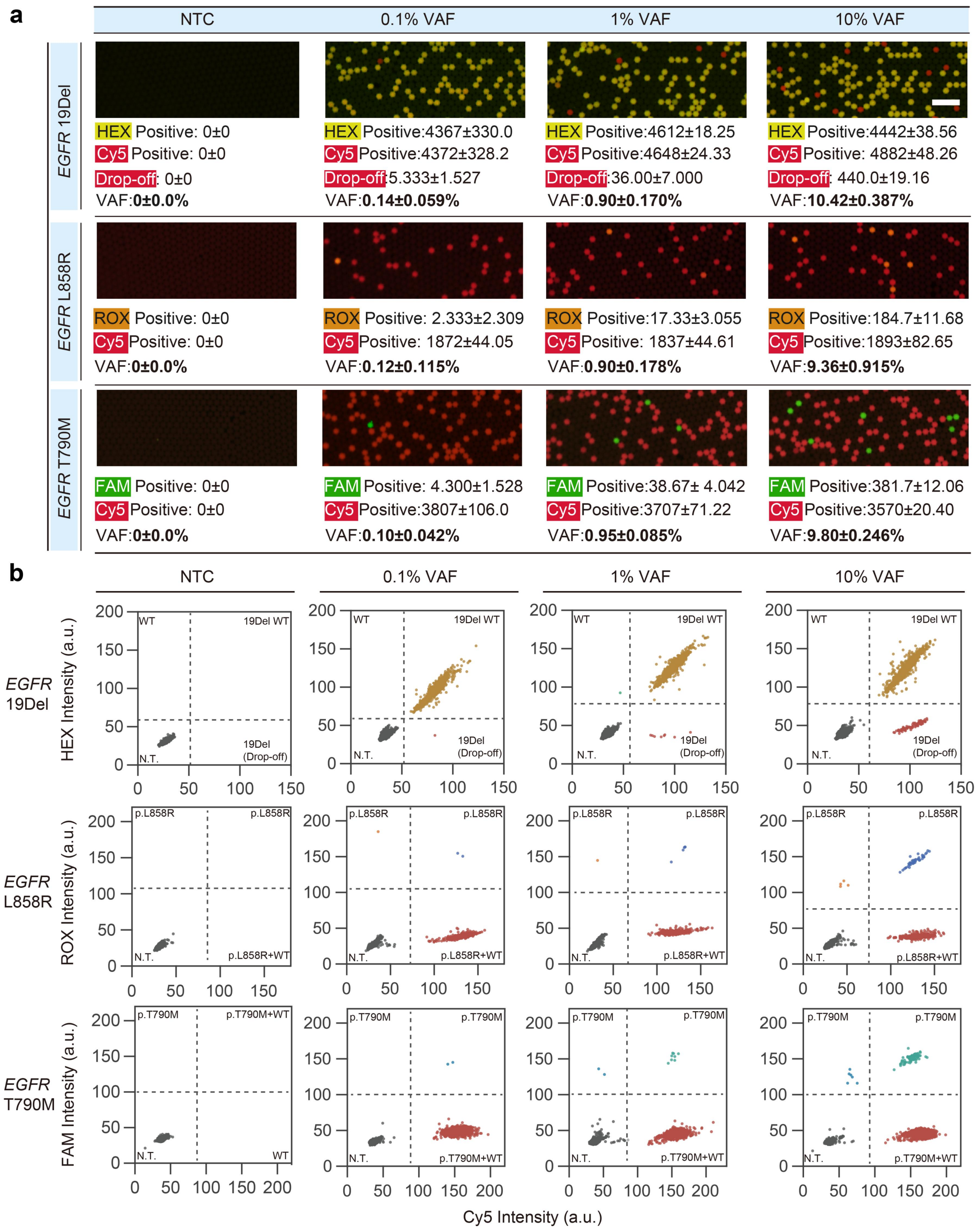

Figure S16. Quadruplex dPCR Assay Validation for *EGFR* Variant Allele Frequency (VAF) Measurement. (a) Fluorescent imaging of samples with varied *EGFR* mutations (T790M, L858R, 19Del) at different VAFs (blank, 0.1%, 1%, 10%), achieved by spiking synthetic mutant plasmids into human DNA. Beneath each image, the mean and standard deviation of positive droplets are provided based on three replicate analyses of 20,000 droplets per assay. Scale bar: 500 µm. (b) Corresponding 2D scatter plots from the quadruplex dPCR assay illustrate the detection efficiency across the range of VAFs: blank, 0.1%, 1%, and 10%. The droplet populations are shown at the four corners of the scatter plot.

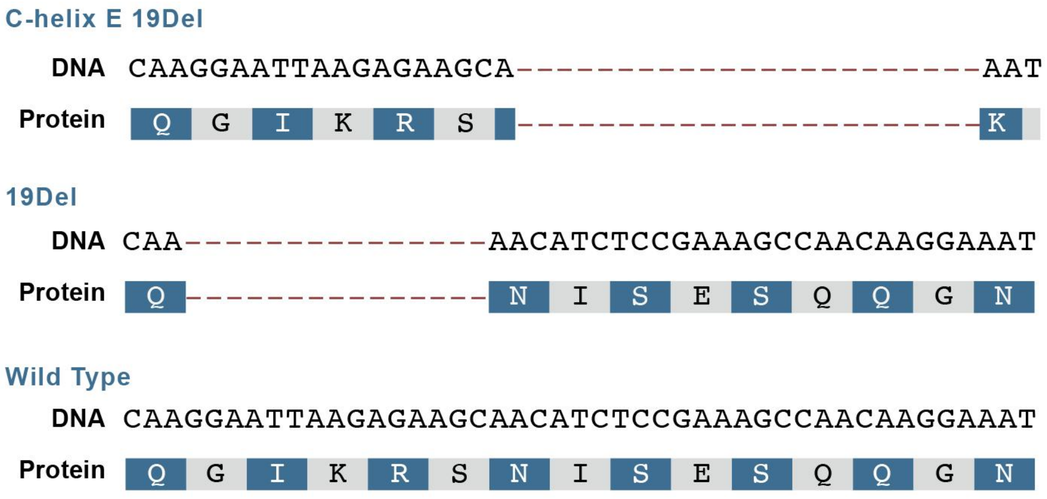

Figure S17. Alignment of the sequencing results of C-helix 19Del, 19Del, and wild type.

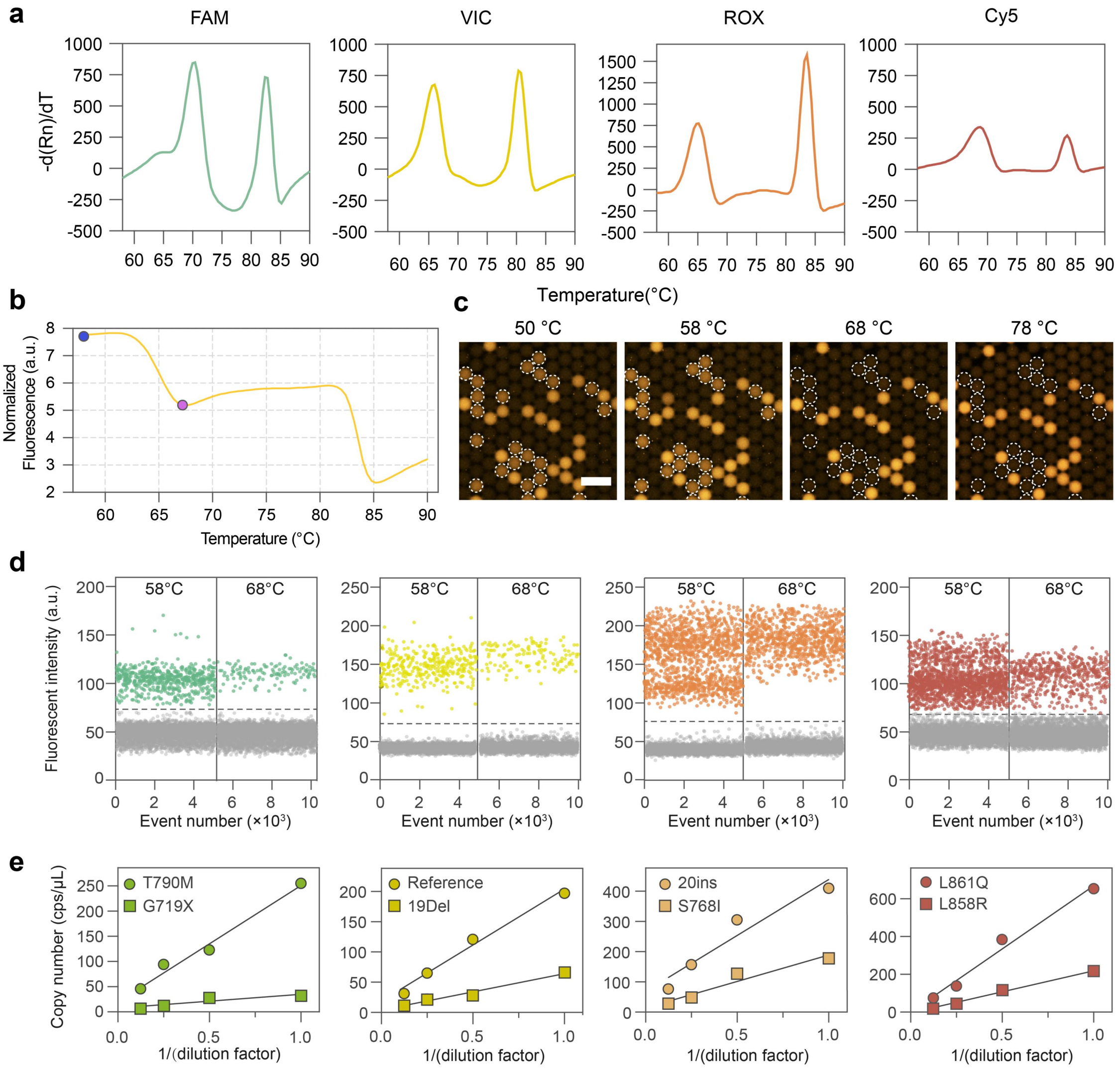

Figure S18. Multicolor Melt Curve Analysis (MCA) and Digital Stepwise Melting Analysis (dSMA) of the High-Multiplex *EGFR* Assay. (a) qPCR MCA of the high-multiplex *EGFR* dSMA assay in four fluorescent channels. Utilizing custom-developed positive control material of *EGFR* mutations, distinct melting peaks were obtained for each channel: 68 °C and 82 °C for FAM, 65 °C and 80 °C for HEX, 65 °C and 82 °C for ROX, as well as 67 °C and 83 °C for Cy5. (b) The dissociation curve was measured by qPCR. The temperatures 58 °C and 68 °C are pivotal for this dSMA assay. (c) Fluorescence images captured at 50 °C, 58 °C, 68 °C, and 78 °C in the ROX channel. White dashed lines encircle droplets that exhibited quenching at 68 °C. Scale bar: 300 μm. (d) 1-D scatter plots of *EGFR* dSMA at 58°C and 68°C, shown in separate fluorescence channels. (e) Linear regression highlights the link between dilution factors and measured concentrations.

Supporting Tables

Table S1. Specification comparison of OsciDrop dPCR system with dedicated dPCR platforms.

Methods	chip-based droplets		microfabricated chambers		OsciDrop
dPCR platforms	QX200/QXONE	Naica	QIAcuity	Absolute Q	This work
Manufacturer	Bio-Rad	Stila	Qiagen	ThermoFisher	Maccura
All-in-one	No/Yes	No	Yes	Yes	Yes
Fully automated	No/Yes	No	Yes	Yes	Yes
Chip-free	No	No	No	No	Yes
Declared partition number per sample	20,000	20,000–30,000	8,400/26,000	20,480	5,000/10,000/20,000
Samples per run	8–96 and 480	12	24 or 96 per plate, up to 8 plates per run	16	24, 48 or 96
Partition volume	0.795 nL	0.43 nL, fixed	0.34 nL, fixed	0.52 nL, fixed	1.0 nL, adjustable to 0.2–5.0 nL without changing pipette tips[1]
Oil	Fluorinated oil	Fluorinated oil	N.A.	Silicone oil	Mineral oil
Thermoblocks	1	1	1–2	1	3
Melting analysis	No	No	No	No	Yes, dSMA
Multiplex capability	2 colors and 4 colors, up to 10-plex based on amplitudes	3 or 6 colors	5 colors	5 colors	6 colors
Sample Recovery	No	Yes	No	No	Yes

Table S2. Advancements in droplet generation: OsciDrop dPCR system vs. previous OsciDrop module.

Platform	Original OsciDrop module[1]	OsciDrop dPCR system (this work)
Schematic diagram of droplet generation module	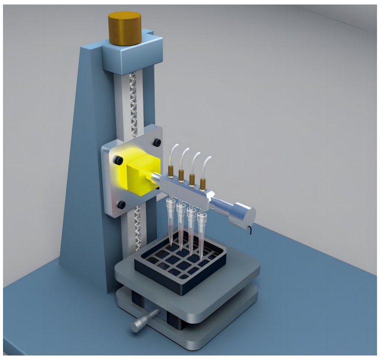	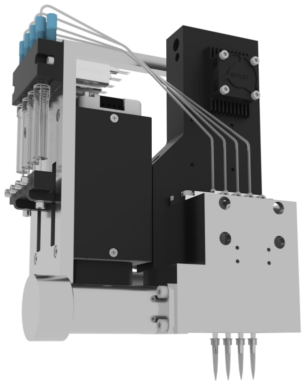
Snapshot from High-speed movie	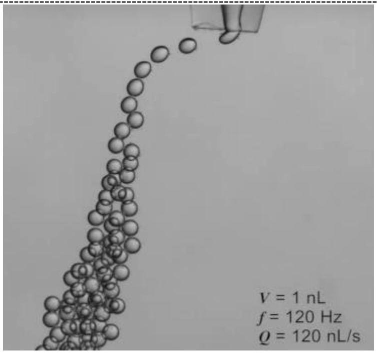	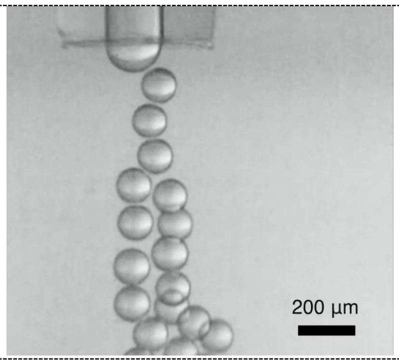
Waveform	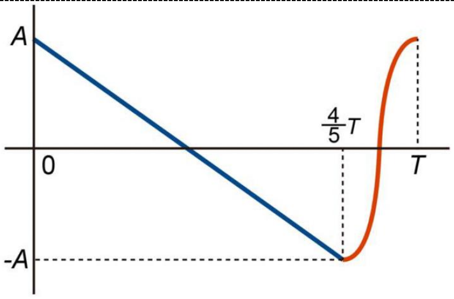	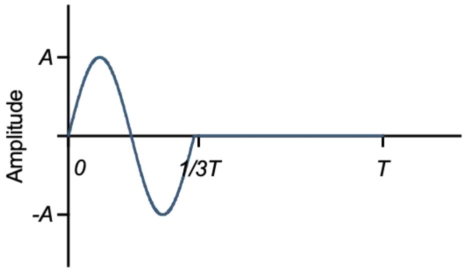
Waveform description	asymmetric	symmetric with periodicity stop
Integration	The pump and controller not integrated.	Fully integrated with a 4-channel pump and controllers in a miniaturized setup.
Amplitude	900 mV	450 mV
Throughput	120 Hz	122 Hz
Submersion depth of the pipette tip	0 ~ 0.3 mm under the oil surface (limited applicability)	0 ~ 0.9 mm under the oil surface (suitable for fully automated systems)
Tip end inner diameter	120 µm	160 µm

Table S3. Synthesized plasmid for *EGFR* variant allele frequency (VAF) quantification assay evaluation.

Plasmid	Sequence
EGFR-2573T	TAGTCACTAACGTTCGCCAGCCATAAGTCCTCGACGTGGAGAGGCTCAGAGCCTGGCATGAACA<br>TGACCCTGAATTCGGATGCAGAGCTTCTTCCCATGATGATCTGTCCCTCACAGCAGGGTCTTCTC<br>TGTTTCAGGGCATGAACTACTTGGAGGACCGTCGCTTGGTGCACCGCGACCTGGCAGCCAGGAA<br>CGTACTGGTGAAAACACCGCAGCATGTCAAGATCACAGATTTTGGGCTGGCCAAACTGCTGGGT<br>GCGGAAGAGAAAGAATACCATGCAGAAGGAGGCAAAGTAAGGAGGTGGCTTTAGGTCAGCCAG<br>CATTTTCCTGACACCAGGGACCAGGCTGCCTTCCCACTAGCTGTATTGTTTAACACATGCAGGGG<br>AGGATGCTCTCCAGACATTCTGGGTGAGCTCGCAGCAGCTGCTGCTGGCAGCTGGGTCCAGCC<br>AGGGTCTCCTGGTAGTGTGAGCCAGAGCTGCTT
EGFR-2573G	TAGTCACTAACGTTCGCCAGCCATAAGTCCTCGACGTGGAGAGGCTCAGAGCCTGGCATGAACA<br>TGACCCTGAATTCGGATGCAGAGCTTCTTCCCATGATGATCTGTCCCTCACAGCAGGGTCTTCTC<br>TGTTTCAGGGCATGAACTACTTGGAGGACCGTCGCTTGGTGCACCGCGACCTGGCAGCCAGGAA<br>CGTACTGGTGAAAACACCGCAGCATGTCAAGATCACAGATTTTGGGCGGGCCAAACTGCTGGGT<br>GCGGAAGAGAAAGAATACCATGCAGAAGGAGGCAAAGTAAGGAGGTGGCTTTAGGTCAGCCAG<br>CATTTTCCTGACACCAGGGACCAGGCTGCCTTCCCACTAGCTGTATTGTTTAACACATGCAGGGG<br>AGGATGCTCTCCAGACATTCTGGGTGAGCTCGCAGCAGCTGCTGCTGGCAGCTGGGTCCAGCC<br>AGGGTCTCCTGGTAGTGTGAGCCAGAGCTGCTT
EGFR-2235_2249del15	GGAGCCCAACAGCTGCAGGGCTGCGGGGGCGTCACAGCCCCCAGCAATATCAGCCTTAGGTGC<br>GGCTCCACAGCCCCAGTGTCCCTCACCTTCGGGGTGCATCGCTGGTAACATCCACCCAGATCAC<br>TGGGCAGCATGTGGCACCATCTCACAATTGCCAGTTAACGTCTTCCTTCTCTCTCTGTCATAGGG<br>ACTCTGGATCCCAGAAGGTGAGAAAGTTAAAATTCCCGTCGCTATCAAAACATCTCCGAAAGCCA<br>ACAAGGAAATCCTCGATGTGAGTTTCTGCTTTGCTGTGTGGGGGTCCATGGCTCTGAACCTCAGG<br>CCCACCTTTTCTCATGTCTGGCAGCTGCTCTGCTCTAGACCCTGCTCATCTCCACATCCTAAATGT<br>TCACTTTCTATGTCTTTCCCTTTCTAGCTCTAGTGGGTATAACTCCCTCCCCTTAGAGACAGCACT<br>GGCCTCTCCCATGCTGGTATCCACCC
EGFR-2235_2249w	GGAGCCCAACAGCTGCAGGGCTGCGGGGGCGTCACAGCCCCCAGCAATATCAGCCTTAGGTGC<br>GGCTCCACAGCCCCAGTGTCCCTCACCTTCGGGGTGCATCGCTGGTAACATCCACCCAGATCAC<br>TGGGCAGCATGTGGCACCATCTCACAATTGCCAGTTAACGTCTTCCTTCTCTCTCTGTCATAGGG<br>ACTCTGGATCCCAGAAGGTGAGAAAGTTAAAATTCCCGTCGCTATCAAGGAATTAAGAGAAGCAA<br>CATCTCCGAAAGCCAACAAGGAAATCCTCGATGTGAGTTTCTGCTTTGCTGTGTGGGGGTCCATG<br>GCTCTGAACCTCAGGCCCACCTTTTCTCATGTCTGGCAGCTGCTCTGCTCTAGACCCTGCTCATC<br>TCCACATCCTAAATGTTCACTTTCTATGTCTTTCCCTTTCTAGCTCTAGTGGGTATAACTCCCTCCC<br>CTTAGAGACAGCACTGGCCTCTCCCATGCTGGTATCCACCC

Table S4. ***EGFR*** **Variant Allele Frequency (VAF) quantification in clinical samples.**

Sample Number	Sex	Age	ARMs-qPCR Qualitative	VAF Quantification					
				19Del		L858R		T790M	
				dPCR	NGS	dPCR	NGS	dPCR	NGS
N1	Female	46	N.D.	13.258%	N.A.	N.D.	N.A.	N.D.	N.A.
N2	Female	54	N.A.	N.D.	N.D.	N.D.	N.D.	N.D.	N.D.
N3	Female	34	N.D.	N.D.	N.A.	N.D.	N.A.	N.D.	N.A.
N4	Male	67	N.D.	N.D.	N.A.	N.D.	N.A.	N.D.	N.A.
N5	Male	77	N.D.	N.D.	N.A.	N.D.	N.A.	N.D.	N.A.
N6	Male	63	N.D.	N.D.	N.A.	N.D.	N.A.	N.D.	N.A.
N7	Male	54	N.D.	N.D.	N.A.	N.D.	N.A.	N.D.	N.A.
N8	Male	67	N.D.	N.D.	N.A.	N.D.	N.A.	N.D.	N.A.
N9	Male	63	N.D.	N.D.	N.A.	N.D.	N.A.	N.D.	N.A.
N10	Male	54	N.D.	N.D.	N.A.	N.D.	N.A.	N.D.	N.A.
N11	Male	72	N.D.	N.D.	N.A.	N.D.	N.A.	N.D.	N.A.
N12	Male	53	N.D.	N.D.	N.A.	N.D.	N.A.	N.D.	N.A.
N13	Male	69	N.D.	N.D.	N.A.	N.D.	N.A.	N.D.	N.A.
N14	Male	81	N.D.	N.D.	N.A.	N.D.	N.A.	N.D.	N.A.
N15	Male	51	N.D.	N.D.	N.A.	N.D.	N.A.	N.D.	N.A.
N16	Male	70	N.D.	N.D.	N.A.	N.A.	N.A.	N.D.	N.A.
N17	Male	71	N.D.	N.D.	N.A.	N.D.	N.A.	N.D.	N.A.
N18	Male	71	N.D.	N.D.	N.A.	N.D.	N.A.	N.D.	N.A.
N19	Male	60	N.D.	N.D.	N.A.	N.D.	N.A.	N.D.	N.A.
N20	Male	74	N.D.	N.D.	N.A.	N.D.	N.A.	N.D.	N.A.
P1	Female	55	19Del&T790M	13.05%	N.A.	N.D.	N.A.	15.03%	N.A.
P2	Female	55	19Del	56.40%	N.A.	N.D.	N.A.	N.D.	N.A.
P3	Female	57	19Del	11.09%	N.A.	N.D.	N.A.	N.D.	N.A.
P4	Male	52	19Del	21.33%	N.A.	N.D.	N.A.	N.D.	N.A.
P5	Male	69	19Del	9.09%	N.A.	N.D.	N.A.	N.D.	N.A.
P6	Male	53	19Del	23.36%	N.A.	N.D.	N.A.	N.D.	N.A.
P7	Male	48	N.A.	19.68%	16.89%	N.D.	N.D.	N.D.	N.D.
P8	Male	65	19Del	13.83%	N.A.	N.D.	N.A.	N.D.	N.A.
P9	Female	70	19Del	14.84%	N.A.	N.D.	N.A.	N.D.	N.A.
P10	Male	66	19Del	33.40%	N.A.	N.D.	N.A.	N.D.	N.A.
P11	Female	52	N.A.	N.D.	N.D.	8.86%	8.12%	N.D.	N.D.
P12	Female	83	L858R	N.D.	N.A.	31.47%	N.A.	N.D.	N.A.
P13	Male	64	N.A.	N.D.	N.D.	49.07%	51.43%	N.D.	N.D.
P14	Female	47	N.A.	N.D.	N.D.	13.51%	12.89%	N.D.	N.D.
P15	Male	49	L858R	N.D.	N.A.	36.33%	N.A.	N.D.	N.A.
P16	Female	76	N.A.	N.D.	N.A.	3.60%	3.74%	N.D.	N.A.
P17	Female	68	N.A.	N.D.	N.A.	7.03%	7.49%	N.D.	N.A.
P18	Male	65	N.A.	N.D.	N.A.	15.50%	16.49%	N.D.	N.A.
P19	Female	67	N.A.	N.D.	N.A.	23.66%	29.59%	N.D.	N.A.
P20	Female	72	N.A.	N.D.	N.D.	21.61%	22.18%	N.D.	N.D.

Note: N.A., not available; N.D., not detected.

Table S5. Target information for the multiplex *EGFR* assay based on digital stepwise melting analysis (dSMA).

Exon	Type	Mutation	Color	T_m	Observed R^2
EGFR 18 exon	G719A	c.2156G>C	FAM	82°C	0.9092
	G719C	c.2155G>T			
	G719S	c.2155G>A			
EGFR 19 exon	p.E746_A750del(1)	c.2235_2249del15	HEX	80°C	0.9735
	p.E746_A750del(2)	c.2236_2250del15			
	p.L747_P753>S	c.2240_2257del18			
	p.E746_T751>I	c.2235_2252>AAT(complex)			
	p.E746_T751del	c.2236_2253del18			
	p.E746_T751>A	c.2237_2251del15			
	p.E746_S752>A	c.2237_2254del18			
	p.E746_S752>V	c.2237_2255>T(complex)			
	p.E746_S752>D	c.2238_2255del18			
	p.L747_A750>P	c.2238_2248>GC(complex)			
	p.L747_T751>Q	c.2238_2252>GCA(complex)			
	p.L747_E749del	c.2239_2247delTTAAGAGAA			
	p.L747_T751del	c.2239_2253del15			
	p.L747_S752del	c.2239_2256del18			
	p.L747_A750>P	c.2239_2248TTAAGAGAAG>C(complex)			
	p.L747_P753>Q	c.2239_2258>CA(complex)			
	p.L747_T751>S	c.2240_2251del12			
	p.L747_T751del	c.2240_2254del15			
	p.L747_T751>P	c.2239_2251>C(complex)			
	p.L747_T751>Q	c.2239_2253>CAA			
	p.E746_S752delinsI	c.2235_2255>AAT			
	p.E746_T751delinsV	c.2237_2252>T			
EGFR 20 exon	p.V769_D770insASV	c.2307_2308insGCCAGCGTG	ROX	65°C	0.9275
	p.D770_N771insSVD	c.2311_2312insGCGTGGACA			
	p.H773_V774insNPH	c.2319_2320insAACCCCCAC			
	p.D770_N771insG	c.2310_2311insGGT			
	p.H773_V774insH	c.2319_2320insCAC			
	T790M	c.2369C>T	FAM	68°C	0.9813
	S768I	c.2303G>T	ROX	82°C	0.9368
EGFR 21 Exon	L858R	c.2573T>G	Cy5	83°C	0.9944
	L861Q	c.2582T>A	Cy5	67°C	0.9823
Reference	Concerved gene	-	HEX	65°C	0.9848

Table S6. Sequences of primers and probes.

Name	Oligo Type	5'-Fluorophore	Sequence	3'-Quencher	Reference
*HER2*_F	Primer	-	TCTTAGACCATGTCCGGGAAA	-	-
*HER2*_R	Primer	-	GAGCCAGCCCGAAGTCTGTA	-	-
*HER2*_P	Probe	FAM	GGAGGATGTGCGGCTCGTAC	MGB	-
*CEP17*_F	Primer	-	TATTTCATTCCTGCAGCCC	-	-
*CEP17*_R	Primer	-	TCCTGCACTGTAACACGT	-	-
*CEP17*_P	Probe	HEX	AGCAGGTCCAGCCCA	MGB	-
*TGFβ3*_F	Primer	-	TCTGAGCCTTCGTTTCCGTAT	-	-
*TGFβ3*_R	Primer	-	CAGAGCACAGGTGAGGGAGC	-	-
*TGFβ3*_P1	Probe	FAM	ACCAGTGGTTCCTGAATGCCAT	BHQ1	-
*TGFβ3*_P2	Probe	Cy5	ACCAGTGGTTCCTGAATGCCAT	BHQ3	-
*RNase P*_F	Primer	-	CAGATTTGGACCTGCGAGC	-	-
*RNase P*_R	Primer	-	GAGCGGCTGTCTCCACAAGT	-	-
*RNase P*_P	Probe	VIC	TTCTGACCTGAAGGCTCTGCG	BHQ1	-
*ERBB2*_Molecular_beacon	Probe	FAM	CGATCGGGAGGATGTGCGGCTCGTACCGATCG	BHQ1	[2]
*N-HER2*_F	Primer	-	CTCATCGCTCACAACCAAGT	-	-
*N-HER2*_R	Primer	-	GGTCTCCATTGTCTAGCACG	-	-
*N-HER2*_P	Probe	ROX	ACCCAGCTCTTTGAGGACAACTATGC	BHQ2	-
*ORF1ab*_F	Primer	-	CCCTGTGGGTTTTACACTTAA	-	-
*ORF1ab*_R	Primer	-	ACGATTGTGCATCAGCTGA	-	
*ORF1ab*_P	Probe	FAM	CCGTCTGCGGTATGTGGAAAGGTTATGG	BHQ1	
*N*_F	Primer	-	GGGGAACTTCTCCTGCTAGAAT	-	
*N*_R	Primer	-	CAGACATTTTGCTCTCAAGCTG	-	
*N*_P	Probe	FAM	TTGCTGCTGCTTGACAGATT	BHQ1	
*35S*_F	Primer	-	GCCTCTGCCGACAGTGGT	-	[3,4]
*35S*_R	Primer	-	AAGACGTGGTTGGAACGTCTTC	-	
*35S*_P	Probe	HEX	CAAAGATGGACCCCCACCCACG	BHQ1	
*T-nos-180*_F	Primer	-	CATGTAATGCATGACGTTATTTATG	-	
*T-nos-180*_R	Primer	-	TTGTTTTCTATCGCGTATTAAATGT	-	
*T-nos-180*_P	Probe	FAM	ATGGGTTTTTATGATTAGAGTCCCGCAA	BHQ1	
*Le1 B*_F	Primer	-	CCAGCTTCGCCGCTTCCTTC	-	
*Le1 B*_R	Primer	-	GAAGGCAAGCCCATCTGCAAGCC	-	
*Le1 B*_P	Probe	CY5	CTTCACCTTCTATGCCCCTGACAC	BHQ2	
*cp4-GT73*_F	Primer	-	GGGATGACGTTAATTGGCTCTG	-	
*cp4-GT73*_R	Primer	-	GGCTGCTTGCACCGTGAAG	-	
*cp4-GT73*_P	Probe	ROX	CACGCCGTGGAAACAGAAGACATGACC	BHQ2	

Table S7. List of thermocycling conditions and fluorescence imaging parameters for assays used in this work.

Assay/kit Name	Thermocycling Condition	Fluorescence imaging parameters
OsciDrop dPCR Starter kit (Maccura)	Step 1: 95°C, 5 min<br>Step 2: 94°C, 20 sec<br>Step 3: 56°C, 1 min<br>Step 2 to 3: 45 cycles<br>Hold: 25 °C	FAM: 3 sec, 20 gain<br>HEX: 2 sec, 20 gain<br>ROX: 2 sec, 20 gain<br>Cy5: 2 sec, 20 gain
dPCR instrument copy number calibration (NIM)	Step 1: 95°C, 8 min<br>Step 2: 94°C, 30 sec<br>Step 3: 56°C, 1 min 15 s<br>Step 2 to 3: 45 cycles<br>Hold: 25 °C	FAM: 3 sec, 20 gain<br>HEX: 2 sec, 20 gain<br>ROX: 2 sec, 20 gain
SARS-CoV-2 *ORF1ab* &*E*	Step 1:50°C, 30 min<br>Step 2:95°C, 10 min<br>Step 3: 94°C, 30 sec<br>Step 4: 56°C, 1 min<br>Step 3 to 4: 45 cycles<br>Hold: 25 °C	FAM: 3 sec, 20 gain<br>ROX: 2 sec, 20 gain
SARS-CoV-2 *N*	Step 1:50°C, 30 min<br>Step 2:95°C, 10 min<br>Step 3: 94°C, 30 sec<br>Step 4: 56°C, 1 min<br>Step 3 to 4: 45 cycles<br>Hold: 25 °C	FAM: 3 sec, 20 gain
Human gDNA concentration measurement (Maccura)	Step 1: 95°C, 5 min<br>Step 2: 94°C, 20 sec<br>Step 3: 56°C, 1 min<br>Step 2 to 3: 45 cycles<br>Hold: 25 °C	FAM: 3 sec, 20 gain<br>HEX: 2 sec, 20 gain<br>ROX: 2 sec, 20 gain<br>Cy5: 2 sec, 20 gain
HER2:CEP17 CNV assessment kit (Maccura)	Step 1: 95°C, 5 min<br>Step 2: 94°C, 20 sec<br>Step 3: 58°C, 1 min<br>Step 2 to 3: 40 cycles<br>Hold: 25 °C	FAM: 3 sec 20 gain<br>HEX: 3 sec, 20 gain
quadruplex assay for *EGFR* VAF quantification (Maccura)	Step 1: 95°C, 5 min<br>Step 2: 94°C, 20 sec<br>Step 3: 56°C, 1 min<br>Step 2 to 3: 45 cycles<br>Hold: 25 °C	FAM: 3 sec, 20 gain<br>HEX: 2 sec, 20 gain<br>ROX: 2 sec, 20 gain<br>Cy5: 2 sec, 20 gain
dSMA *EGFR* variants profiling (Maccura)	Step 1: 95°C, 1 min<br>Step 2: 95°C, 15sec<br>Step 3: 57°C, 40 sec<br>Step 2 to 3: 8 cycles<br>Step 4: 95°C, 15 sec<br>Step 5: 69°C, 40 sec<br>Step 4 to 5: 4 cycles<br>Step 6: 95°C, 15 sec<br>Step 7: 69°C, 40 sec	Stage 1: 68°C Hold<br>FAM: 3 sec, 20 gain<br>HEX: 2.5 sec, 20 gain<br>ROX: 2 sec, 20 gain<br>Cy5: 3 sec, 20 gain<br>Stage 2: 58°C Hold<br>FAM: 3 sec, 20 gain<br>HEX: 2.5 sec, 20 gain<br>ROX: 2 sec, 20 gain

	Step 6 to 7: 5 cycles<br>Step 8: 68°C, 2 min	Cy5: 3 sec, 20 gain
Certified reference material quantification (Bio-Rad)	Step 1: 95°C, 10 min<br>Step 2: 94°C, 30 sec<br>Step 3: 60°C, 1 min<br>Step 2 to 3: 40 cycles	N.A.
Certified reference material quantification (QIAcuity)	Step 1: 95°C, 5 min<br>Step 2: 95°C, 15 sec<br>Step 3: 60°C, 30 sec<br>Step 2 to 3: 40 cycles	FAM: 0.5 sec, 6 gain<br>HEX: 0.5 sec, 6 gain
Certified reference material quantification (OsciDrop)	Step 1: 95°C, 5 min<br>Step 2: 94°C, 30 sec<br>Step 3: 60°C, 1 min<br>Step 2 to 3: 45 cycles	58°C Hold<br>FAM: 3 sec, 20 gain<br>HEX: 3 sec, 20 gain
Certified reference material quantification (Absolute Q)	Step 1: 96°C, 10 min<br>Step 2: 96°C, 5 sec<br>Step 3: 60°C, 15 sec<br>Step 2 to 3: 40 cycles	N.A.
Certified reference material quantification (Naica)	Step 1: 95°C, 5 min<br>Step 2: 95°C, 30 sec<br>Step 3: 60°C, 30 sec<br>Step 2 to 3: 45 cycles	FAM: 0.3 sec<br>HEX: 0.6 sec

Legend to Supporting Movies

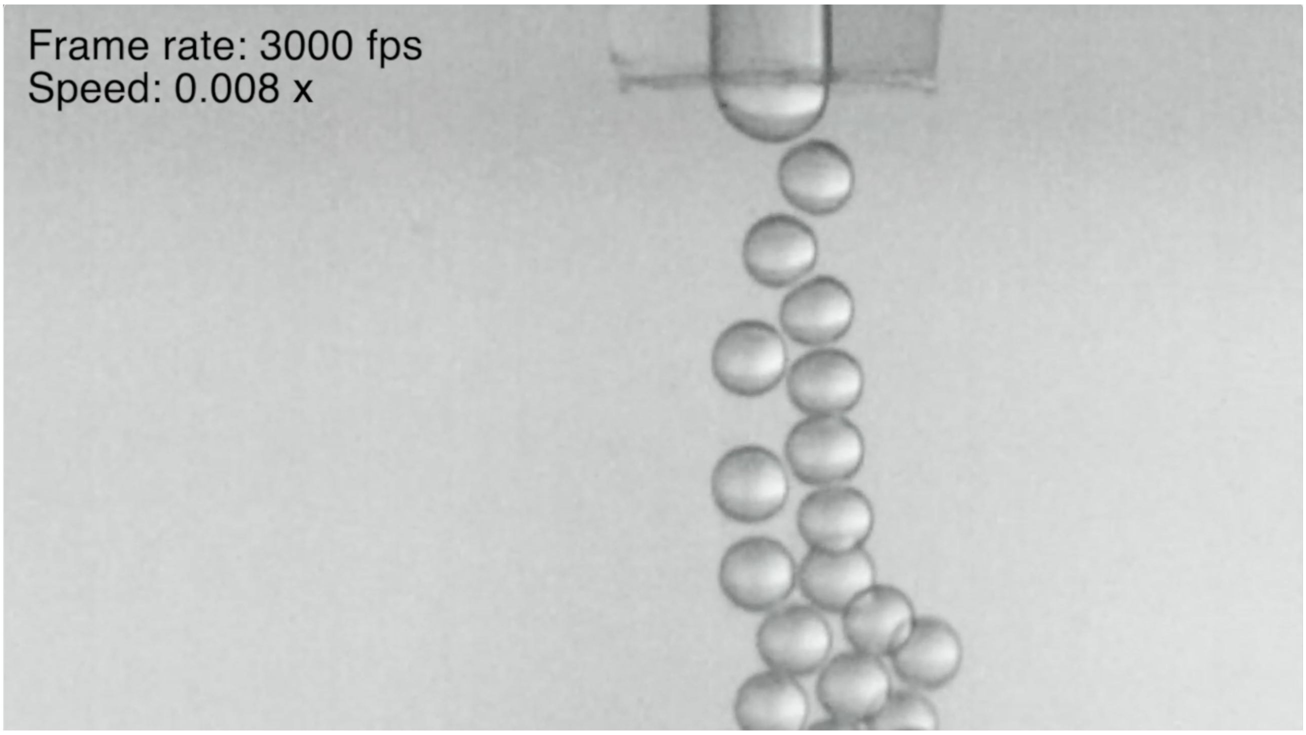

Movie S1. Generating deterministic 1-nL droplets through symmetrical oscillation with periodicity stops. This video demonstrates how symmetrical oscillation of the pipette tip, punctuated by periodic stops, precisely produces droplets with a uniform volume of 1 nanoliter.

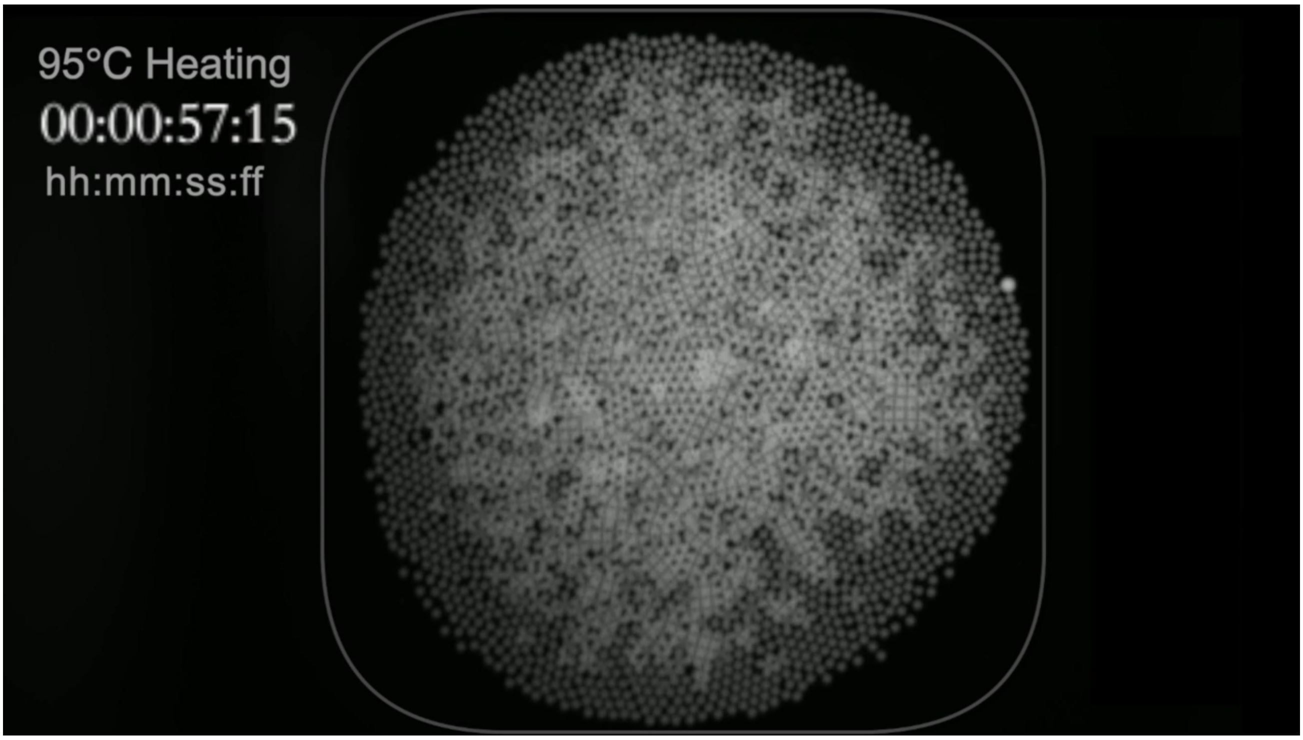

Movie S2. Dynamic Arrange of planar monolayer droplet arrays (PMDAs) in a microwell. This movie captures the dynamic arrangement of 1-nL droplets into a PMDA. Observe the swift spreading of droplets during the 95 °C denaturation step, followed by the formation of the PMDA during the initial annealing cycle, which occurs between 50 °C and 65 °C.

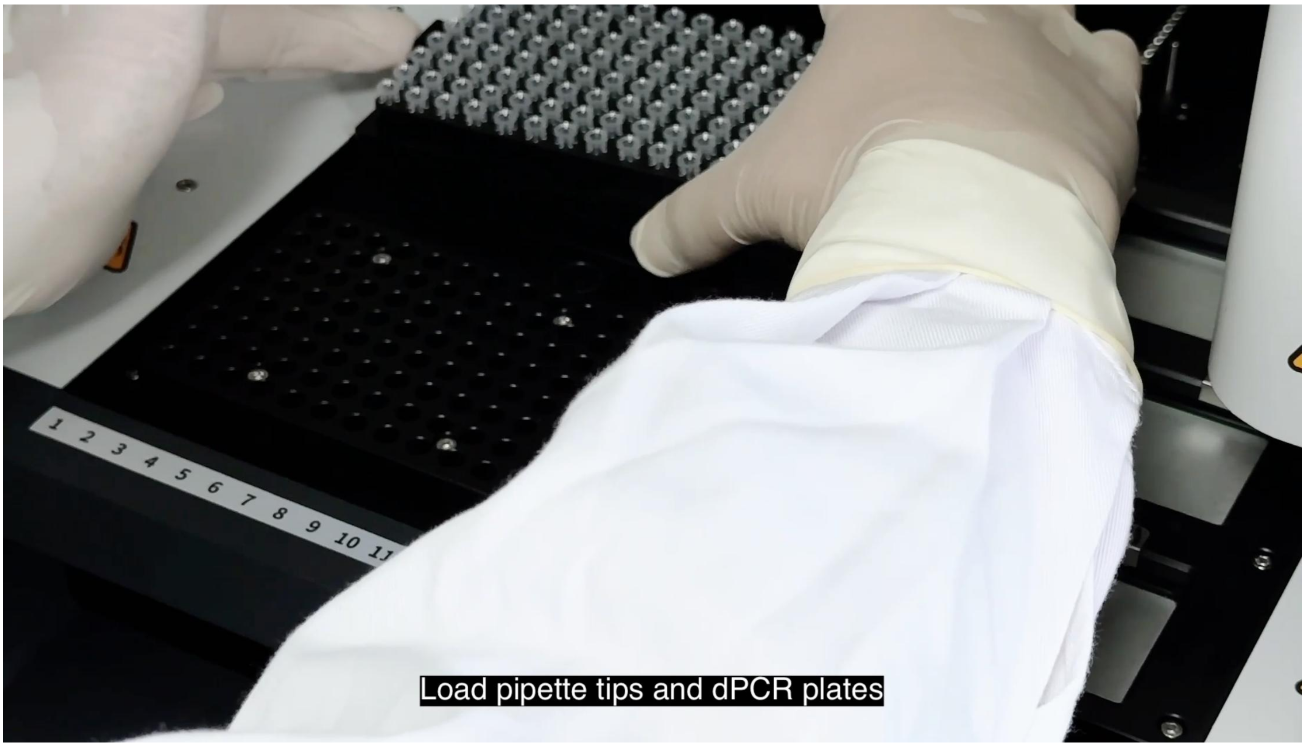

Movie S3. Comprehensive workflow demonstration of the OsciDrop digital PCR system. This video provides a detailed demonstration of the OsciDrop digital PCR system, covering each workflow step. It starts with the preparation phase, including loading pipette tips and dPCR plates, followed by checking the carrier and droplet generation oil bottles with system monitoring consumption. The process continues with the loading of sample tubes and lid removal. Highlighting the system's ease of use, the setup for the experiment configuration is brief. Once the process is initiated, a countdown timer displays the remaining time until completion. The video concludes with the system's fully automated data analysis and report generation, showcasing the efficiency and user-friendliness of the OsciDrop system in conducting dPCR detection and analysis.

Supporting Text.

Text S1. SLAS-compliant dPCR liquid handling and droplet printing optimization.

In designing our pipette tips and dPCR plates, we rigorously followed the Society for Laboratory Automation and Screening (SLAS, USA) standards, ensuring compatibility for high-throughput screening and automation. Our compliance with SLAS guidelines includes the following specifics:

1) **32-well dPCR plate design:** Our 32-well dPCR plate, measuring 44 mm x 89 mm, aligns with approximately one-third the size of a standard 96-well plate, conforming to the ANSI/SLAS 1-2004 (R2012) footprint standard. The wells are arranged in an 8 x 4 matrix, each placed at a standardized center-to-center spacing of 9 mm (Figure S2c), ensuring uniformity in well distribution.
2) **Uniform well depth and volume:** We have standardized the depth and volume across all wells on our dPCR plate. This uniformity is critical for consistent droplet array printing and reliable assay results.
3) **Plate height and enhanced stackability:** The 32-well dPCR plate features a height of 15 mm and is specifically designed to be compatible with automated handling systems. Structural enhancements facilitate stable and aligned stacking, reducing the risk of misalignment or accidental displacement.
4) **Optimized pipette tip and pipettor interface:** The pipette tips are engineered for a secure fit with the pipettor, minimizing oil leakage and preventing dislodging, which is vital for precise liquid handling.
5) **Efficient pipette tip packaging:** Packaged in racks of 96 tips, our pipette tips are designed for space-efficient storage and stability, enabling easy stacking and handling in laboratory settings.
6) **Accessible pipette tip design:** The packaging of the pipette tips is designed for ease of access to individual tips, while preserving the sterility and integrity of the remaining tips in the rack.

To create planar monolayer droplet arrays (PMDAs) with minimal droplet overlapping or unoccupied microwell areas, we employ a method that generates nanoliter droplets of deterministic volume and consistent number. This results in a tightly packed array within the microwell boundaries. The precision of partition volume (V_p) forms the foundation for accurate nucleic acid quantification using digital PCR (dPCR). In comparison, existing dPCR platforms often experience systematic droplet volume deviations due to microfabrication imperfections, fluid viscosities, and surface tension variations[5]. We recently demonstrated that our OsciDrop technique can precisely define droplet volume and number by adjusting parameters like oscillation frequency, flow rate, and dispensing volume. This ensures controlled droplet volume and number with minimal variations[1].

However, our previous droplet utilization rate was limited to 94.02%, with fluctuations in microwell occupancy due to overlapped droplets, unoccupied areas within PMDAs, and the occasional presence of large droplets. These issues could be attributed to thermal volume fluctuation and mechanical backlash from the liquid dispenser's syringe pumps. To enhance droplet utilization in our fully automated dPCR system, we introduced an optimized droplet printing process as outlined below (Figure S2a-c):

1) Aspirate an additional 300-nL sample and the total volume of droplets, using pipette tips mounted on the liquid dispenser. For example, to generate 20,000 1-nL droplets, aspirate 20.3 μL of the sample;
2) Pre-inject 100-nL samples using the pipette tips into waste reservoirs adjacent to the edge of the dPCR plate (Figure S2c);
3) Segmentize 20 μL samples into 20,000 1-nL droplets using the OsciDrop droplet printer in four microwells to self-assemble PMDAs;
4) Purge 500 nL of liquid through the pipette tip into waste reservoirs, evacuating residual samples (with a nominal volume of 200 nL) and reducing contamination risk when detaching used pipette tips and attaching new ones.

The optimized droplet generation process ensures superior droplet uniformity and sample utilization. Specifically, adopting the "low-dead-volume" liquid handling strategy to generate 20,000 nanoliter droplets wasted only 300 nL of samples. Surprisingly, the new process achieved a higher droplet utilization rate of 97.77% (Figure S3c). Importantly, this precise assembly of PMDAs contributes to increased sample utilization and the expansion of the confidence interval (CI) and the upper limit of the measurement according to the Poisson distribution (Figure S4).

Text S2. Stepwise melting temperature imaging of droplets improves the droplet identification for molecular beacon-based dPCR assays.

The OsciDrop dPCR system provides an innovative approach for in situ fluorescent imaging of temperature-sensitive reactions by adjusting the thermocycler's temperature settings. As mentioned earlier, the fluorescent imaging technique requires a certain baseline level of background intensity to discern negative reaction units. It becomes challenging when working with assays that exhibit low background fluorescent intensity, such as those involving molecular beacons; the traditional solution often involves incorporating a reference dye, which occupies one of the fluorescent channels and limits the system's multiplexing capacity.

In contrast, the OsciDrop dPCR system offers the flexibility to perform imaging at both room temperature and an elevated temperature corresponding to the melting temperature, enabling the opening of the stem-loop structure of the beacon. Consequently, this approach enhances the fluorescent intensity of negative droplets when imaging at an elevated temperature, thereby facilitating more accurate droplet identification. Similarly, when imaging at room temperature, the efficient binding of the beacon enables the identification of positive droplets with high fluorescent intensity. If we combine the data acquired from different temperatures, we'll significantly improve the identification of both positive and negative droplets, resulting in digital measurements with enhanced precision.

We applied this method using a molecular beacon probe from previous research.[2] Imaging at 25°C represented room temperature conditions, 50°C for elevated temperature, and 70°C to simulate positive signals. The results showed a 50% improvement in droplet recognition rate without affecting the signal-to-noise ratio (Fig. 1j, Figure S9). Elevated temperature imaging specifically improved the identification of negative droplets, and crucially, the fluorescence intensity of these droplets at this temperature remained significantly lower than positive signals, ensuring accurate threshold determination for differentiating between negative and positive droplets.

Text S3. Image processing with a machine learning algorithm.

We devised a machine learning algorithm for digital image processing using Python, with the TensorFlow and OpenCV frameworks at its core. This algorithm harnesses two lightweight deep neural networks (DNNs): U-Net for effective droplet segmentation and MobileNet for accurately identifying valid droplets. To train these networks, we assembled two datasets, each meticulously labeled and randomly divided into training, validation, and testing subsets at an 8:1:1 ratio.

Text S4. Quantification of nucleic acid reference materials (RMs).

Digital PCR has found extensive utility in quantifying nucleic acid reference materials (RMs) by determining the number of target molecules per reaction volume through Poisson distribution[6]. Nevertheless, droplet-based digital PCR often contends with batch-to-batch variations in droplet sizes. On the contrary, chamber-based digital PCR leans on precise microfabrication and chamber size deviations introduced through bonding.

To evaluate the performance of the OsciDrop digital PCR in precise measurements of nucleic acid RMs, we determined the concentration of various RMs, including DNA plasmid, RNA, and human

genomic DNA (gDNA). Our assessment encompassed comparing the results against the standard values and uncertainties provided by the National Institute of Metrology (NIM).

For the quantification of human genomic DNA (gDNA), we employed a 4-color assay measuring *TGF-β3* (FAM, Cy5), *RNase P* (VIC), and *N-HER2* (ROX) simultaneously, involving a 4-fold serial dilution of the gDNA RM (Figure S12). The results demonstrated robust R^2 values for *TGF-β3*, *RNase P*, and *N-HER2*, specifically 0.9989, 0.9985, and 0.9984, respectively. Notably, the quantification errors were within ± 10% for concentrations spanning 31 to 10,000 copies/μL.

We assessed the viability of a one-step reverse transcription digital PCR (RT-dPCR) assay for quantifying RNA samples using the SARS-CoV-2 RNA RM, encompassing *ORF1ab*, *E*, and *N* genes. Table S2 provides the primer and probe sequences for the *ORF1ab* and *N* dPCR assays, while the *E* gene dPCR assay was sourced from the RM. A range of 5-fold dilutions of the RNA RM was prepared. The RT-dPCR outcomes showcased a highly linear relationship with the input RNA concentration ($R^2 > 0.99$), spanning 0.9 to 4.2 lg copies/reaction (Figure S13). Notably, the limit of detection (LOD) for the *ORF1ab*, *E*, and *N* genes were 0.985 ± 0.033, 0.931 ± 0.016, and 0.926 ± 0.012 lg copies/reaction, respectively (Figure S14a). To evaluate accuracy, we calculated the quantification error, which consistently remained within the confidence interval (CI) and uncertainty intervals at concentrations of 1.4 to 4.2 lg copies/reaction (Figure S14b). Moreover, the quantification consistency for the SARS-CoV-2 RM was assessed across three independent plates, yielding CV values of 4.67%, 2.53%, and 4.98% for the *ORF1ab*, *E*, and *N* genes, respectively.

Supporting References

[1] S. Ye, C. Li, X. Zheng, W. Huang, Y. Tao, Y. Yu, L. Yang, Y. Lan, L. Ma, S. Bian, Du W, *Anal. Chem.* **2022**, *94*, 2918.

[2] P. J. Lamy, T. Verjat, M. Paye, A. C. Servanton, J. Grenier, P. Leissner, B. Mougin, *Clin. Chem. Lab Med.* **2006**, *44*, 3.

[3] I. Huber, A. Block, D. Sebah, F. Debode, D. Morisset, L. Grohmann, G. Berben, D. štebih, M. Milavec, J. žel, U. Busch, *J. Agric. Food Chem.* **2013**, *61*, 10293.

[4] T. Demeke, M. Holigroski, M. Eng, J. Xing, *Food Control* **2016**, *68*, 105.

[5] S. L. Anna, N. Bontoux, H. A. Stone, *Appl. Phys. Lett.* **2003**, *82*, 364.

[6] B. Vogelstein, K. W. Kinzler, *Proc. Natl. Acad. Sci. U. S. A.* **1999**, *96*, 9236.
